# Identification of ZBTB18 as a novel colorectal tumor suppressor gene through genome-wide promoter hypermethylation analysis

**DOI:** 10.1186/s13148-021-01070-0

**Published:** 2021-04-23

**Authors:** Sarah Bazzocco, Higinio Dopeso, Águeda Martínez-Barriocanal, Estefanía Anguita, Rocío Nieto, Jing Li, Elia García-Vidal, Valentina Maggio, Paulo Rodrigues, Priscila Guimarães de Marcondes, Simo Schwartz, Lauri A. Aaltonen, Alex Sánchez, John M. Mariadason, Diego Arango

**Affiliations:** 1 Group of Biomedical Research in Digestive Tract Tumors, CIBBIM-Nanomedicine, Vall d’Hebron University Hospital, Research Institute (VHIR), Universitat Autònoma de Barcelona, Passeig Vall d’Hebron, 119-129, 08035 Barcelona, Spain; 2 Group of Drug Delivery and Targeting, CIBBIM-Nanomedicine, Vall d’Hebron University Hospital, Research Institute (VHIR), Universitat Autònoma de Barcelona, Passeig Vall d’Hebron, 119-129, 08035 Barcelona, Spain; 3 CIBER de Bioingeniería, Biomateriales Y Nanomedicina (CIBER-BBN), Madrid, Spain; 4 Department of Medical Genetics, Medicum, University of Helsinki, Biomedicum Helsinki, 00290 Helsinki, Finland; 5 Departament d’Estadísitica, Facultat de Biologia, Universitat de Barcelona, 08028 Barcelona, Spain; 6 Olivia Newton-John Cancer Research Institute, Heidelberg, VIC 3084 Australia; 7 School of Cancer Medicine, La Trobe University, Melbourne, 3086 Australia; 8 Group of Molecular Oncology, IRBLleida, 25198 Lleida, Spain

**Keywords:** Colorectal cancer, Zinc finger, ZBTB18, Methylation, Tumor suppressor

## Abstract

**Background:**

Cancer initiation and progression are driven by genetic and epigenetic changes. Although genome/exome sequencing has significantly contributed to the characterization of the genetic driver alterations, further investigation is required to systematically identify cancer driver genes regulated by promoter hypermethylation.

**Results:**

Using genome-wide analysis of promoter methylation in 45 colorectal cancer cell lines, we found that higher overall methylation levels were associated with microsatellite instability (MSI), faster proliferation and absence of *APC* mutations. Because epigenetically silenced genes could represent important oncogenic drivers, we used mRNA expression profiling of colorectal cancer cell lines and primary tumors to identify a subset of 382 (3.9%) genes for which promoter methylation was negatively associated with gene expression. Remarkably, a significant enrichment in zinc finger proteins was observed, including the transcriptional repressor ZBTB18. Re-introduction of ZBTB18 in colon cancer cells significantly reduced proliferation in vitro and in a subcutaneous xenograft mouse model. Moreover, immunohistochemical analysis revealed that ZBTB18 is frequently lost or reduced in colorectal tumors, and reduced ZBTB18 expression was found to be associated with lymph node metastasis and shorter survival of patients with locally advanced colorectal cancer.

**Conclusions:**

We identified a set of 382 genes putatively silenced by promoter methylation in colorectal cancer that could significantly contribute to the oncogenic process. Moreover, as a proof of concept, we demonstrate that the epigenetically silenced gene ZBTB18 has tumor suppressor activity and is a novel prognostic marker for patients with locally advanced colorectal cancer.

**Supplementary Information:**

The online version contains supplementary material available at 10.1186/s13148-021-01070-0.

## Background

Colorectal cancer is the third most common cancer worldwide, and although patient prognosis largely depends on tumor stage at the time of diagnosis [1], colorectal cancer represents the second cause of cancer-related death [2]. Progression of colorectal cancer is associated with the accumulation of genetic and epigenetic defects that drive the multistep process from normal intestinal epithelial cells to a metastatic colorectal tumor. The genetic defects driving the tumorigenic process in colorectal cancer have been studied for several decades and include mutations in *APC, KRAS* and *TP53* among other genes [3]. Epigenetic changes, such as DNA methylation or histone modification, modulate gene expression without altering the primary DNA nucleotide sequence. DNA methylation is one of the most ubiquitous epigenetic modifications, and the best-characterized DNA methylation process, involving the addition of a methyl group at the C5 position of the cytosine ring by DNA methyltransferases [4]. Cytosine methylation in the context of CpG dinucleotides is common across the genome of normal human cells, but global hypomethylation has been observed in cancer cells [5]. However, regions rich in CpG dinucleotides, known as CpG islands, are often hypermethylated in tumors [6, 7]. These CpG islands are present in the promoter regions of over 50% of human genes, and changes in the levels of methylation constitute an important mechanism of transcriptional deregulation during tumor progression [6].

Development of colorectal cancer is driven by three major classical mechanisms of genomic instability. Chromosomal instability, resulting in large chromosomal gains and losses, is a common feature in approximately 80% of colorectal tumors and is an important driver of the oncogenic process. An alternative tumorigenic pathway known as ‘CpG island methylator phenotype’ (CIMP) is observed in 10–20% of the tumors, and it is characterized by the widespread hypermethylation of CpG islands throughout the genome [8]. Finally, defects in mismatch repair mechanisms result in the accumulation of mutations mostly in nucleotide repeats and the microsatellite instable (MSI) phenotype observed in 10–20% of the tumors in the colon and rectum [9–11]. Notably, about 70–80% of MSI colorectal tumors can be attributed to hypermethylation of the MLH1 promoter in CIMP cases [12, 13], although CIMP also occurs without MSI [14]. Interestingly, MSI + and CIMP + tumors are associated with proximal tumor location, poor differentiation and BRAF mutations [12] and may arise from serrated adenomas or polyps [13, 15].

Histone modifications also play an important role in the epigenetic regulation of colorectal tumorigenesis. The most common and best-studied histone modifications altering gene expression are acetylation and methylation. Acetylation is catalyzed by histone acetyl transferases (HATs) and counteracts the positive charge on the tails of histones, weakening the electrostatic interaction between DNA and histones and consequently, reducing the compaction of chromatin and enhancing gene expression. Contrary, deacetylation is associated with gene silencing and is controlled by histone deacetylases (HDACs). In turn, histone methylation creates docking sites in the chromatin that can be recognized by transcriptional complexes to activate or repress gene expression. Histone methylation and demethylation are catalyzed by histone methyltransferases (HMTs) and histone demethylases (HDMs), respectively [4].

While the genetic events that drive the tumorigenic process are relatively well understood for colorectal cancer, the epigenetic events and their impact on the transcriptional reprogramming observed in colorectal tumors have not been as extensively characterized. Specifically for DNA methylation, although genome-wide studies have analyzed the genomic distribution of hypermethylated CpGs in colorectal tumors [3, 7], a detailed analysis of the subset of these events that are important for gene expression deregulation during colorectal tumorigenesis is currently lacking. To bridge this gap, in this study we used genome-wide CpG methylation analysis of a panel of 45 colorectal cancer cell lines and 223 primary colorectal tumors available at The Cancer Genome Atlas (TCGA) to investigate the distribution of DNA methylation changes in colorectal cancer [3]. In addition, by integrating methylation data with mRNA expression data we identified a set of 382 genes, enriched in zinc finger proteins, for which high levels of promoter methylation was associated with reduced gene expression. As a proof of concept, we demonstrate that ZBTB18, a zinc finger transcriptional repressor that is frequently methylated in colorectal tumors, has tumor suppressor activity and is a novel prognostic factor in colorectal cancer.

## Results

### Genome-wide analysis of CpG methylation in colorectal cancer cell lines

**Fig. 1:**
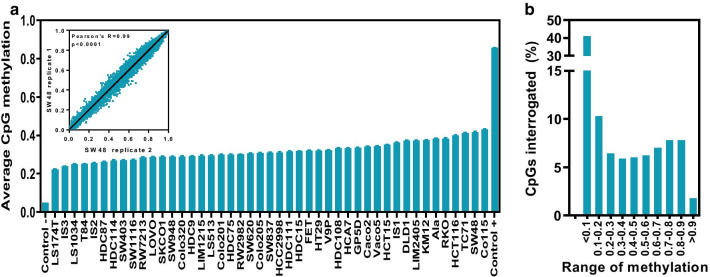
CpG methylation in colorectal cancer cell lines. **a** The average (± SEM) methylation (beta-value) of all the 27,578 CpGs interrogated is shown in a panel of 45 colorectal cancer cell lines. Control − : whole genome amplification with GenomiPhi, GE Healthcare; Control + : in vitro methylated with SssI. Inset shows the correlation between methylation measurements for all CpGs in two replicates of SW48. **b** Histogram showing the percentage of the CpG dinucleotides interrogated that have average methylation levels across the 45 cell lines in the indicated intervals

To investigate genome-wide promoter methylation levels and possible associations with frequent genetic changes observed during colorectal tumorigenesis, the methylation of 27,578 CpG dinucleotides in 14,495 genes was assessed in a panel of 45 colorectal cancer cell lines using an Infinium HumanMethylation27 BeadChip platform (Illumina). A quantitative measurement of the methylation levels (beta values) in each of the 27,578 CpGs was obtained. Global methylation values ranged from 0 (unmethylated) to 1 (fully methylated). As expected, low levels of methylation were observed in an unmethylated control (mean methylation 0.05) and an in vitro methylated sample showed the highest methylation levels (mean methylation 0.86; Fig. 1a). Moreover, independent determinations of genome-wide methylation in SW48 cells demonstrated excellent correlation (inset in Fig. 1a), and independent bisulfite sequencing analysis of selected promoters in a subset of cell lines was in perfect agreement with the HumanMethylation27 BeadChip determinations obtained (Additional file 1: Fig. S1).

The average methylation across all 27,578 CpGs in the 45 colorectal cancer cell lines investigated showed large variability, ranging from 0.22 to 0.43 (Fig. 1a), indicating that cancer cells derived from colorectal tumors can widely vary in their overall levels of methylation. Next, we interrogated global methylation in 233 primary tumors analyzed by The Cancer Genome Atlas (TCGA) project for which the same HumanMethylation27 platform was used [3]. The variability observed across patients’ primary tumors ranged from 0.19 to 0.35 (Additional file 1: Fig. S2a), indicating that the global DNA methylation levels in cell lines reflect that of primary tumors. When the levels of methylation of individual CpGs was considered, the majority (50.2%) showed low average methylation across the panel of 45 colorectal cancer cell lines (< 0.2 methylation), while over 30% of the CpGs showed average methylation levels above 0.5 (Fig. 1b), which again was similar to the distribution observed in primary colorectal tumors (Additional file 1: Fig. S2b).

**Fig. 2:**
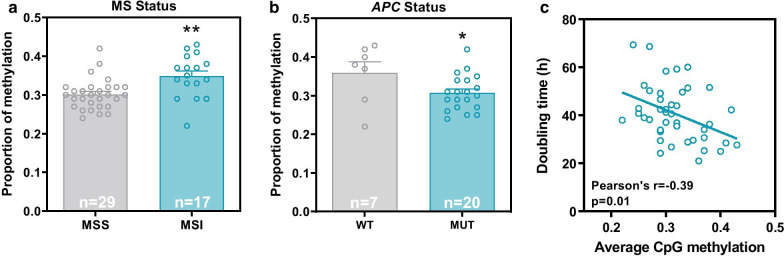
Associations between the average levels of methylation and molecular features of colorectal cancer cell lines. Higher levels of CpG methylation were associated with microsatellite instability (MSI status) (**a**), wild-type *APC* status (**b**) and faster growth (i.e., shorter doubling time) (**c**) in the panel of 45 colorectal cancer cell lines. Student’s t test *p < 0.05 and **p < 0.01

In agreement with the reported association between colorectal tumors with microsatellite instability (MSI) and the CpG methylator phenotype (CIMP) [10], the overall levels of methylation in the panel of cell lines used in our study were significantly higher in MSI cell lines and primary tumors compared to the respective microsatellite stable (MSS) counterpart (Fig. 2a and Additional file 1: Fig. S3a). In addition, the average levels of methylation across the 27,578 CpGs investigated were significantly lower in cell lines and primary tumors with *APC* mutations compared to cell lines and tumors without mutations in this important tumor suppressor gene (Fig. 2b and Additional file 1: Fig. S3b). Primary tumors with *BRAF* mutations had significantly higher levels of methylation than *BRAF* wild-type tumors, while *TP53* mutant tumors had lower levels of methylation than wild-type *TP53* tumors, although these differences did not reach statistical significance in cell lines (Additional file 1: Fig. S3c–d and g–h). No associations were observed between the average methylation levels and mutations in other frequently mutated driver genes in colorectal cancer, including *KRAS* or *PIK3CA* (Additional file 1: Fig. S3e–f and i–j). In addition, we also observed a significant association between higher methylation levels and faster growth rate across the cell lines (Fig. 2c), suggesting that higher levels of promoter CpG methylation may contribute to increased proliferation rates.

### Associations between promoter methylation and gene expression in colorectal cancer

Although it is widely accepted that aberrant CpG methylation is a frequent event during the oncogenic process and that it plays an important role in the transcriptional regulation of many genes, a detailed list of genes whose transcription is frequently regulated by CpG methylation in colorectal cancer cells is currently lacking. To this end, we used microarray analysis to assess the levels of expression of 11,359 (78.47%) of the 14,475 promoters interrogated in the HumanMethylation27 BeadChips in a subset of 30 colorectal cancer cell lines.

This analysis identified 712 genes (6.3%) with a significant negative correlation (FDR adjusted Spearman’s *p* < 0.2) between promoter methylation and mRNA expression in this panel of 30 colorectal cancer cell lines and is therefore candidate genes that may be epigenetically silenced in colorectal cancer cells (Additional file 1: Fig. S4). In addition, we identified 28 genes whose expression was not significantly correlated with the levels of methylation (FDR adjusted Spearman’s *p* > 0.2), but showed a nonlinear negative association between expression and methylation levels (see Methods section; Additional file 1: Fig. S5). In total, therefore, 740 genes (6.5%) were identified as candidate genes whose expression may be regulated by promoter methylation in colorectal cancer cells.

The relative levels of expression of selected genes were independently confirmed by qPCR (Additional file 1: Fig. S6). In addition, increased mRNA expression following treatment with the demethylating agents 5-aza-2′-deoxycytidine (decitabine) was demonstrated for a subset of 9 of these genes in cell lines showing low endogenous expression and high CpG promoter methylation (Additional file 1: Fig. S7), suggesting a direct regulation of gene expression by promoter hypermethylation for most of these genes.

Although the analysis of genes regulated by promoter methylation in primary colorectal tumor samples is complicated by the presence of varying proportions of non-tumor cells, we used the cohort of 223 primary colorectal tumors from the TCGA repository [3] to further validate the association observed between promoter hypermethylation and reduced gene expression. Of the 740 genes identified as potentially regulated by promoter methylation in the colorectal cancer cell lines, expression and methylation data in the primary tumors were available from 653 genes (88.2%). A significant negative correlation (FDR adjusted Spearman’s *p* < 0.2) between expression and methylation was observed for 382 of these 653 genes (58.5%; see Additional file 1: Table S2), identifying a core set of genes potentially silenced by promoter methylation in colorectal cancer.

### Methylation outside CpG islands and gene expression

DNA methylation within CpG islands and flanking regions (‘shores’) is known to be closely linked to transcriptional repression [7]. Accordingly, we found that the majority of genes that are potentially silenced epigenetically in colorectal cancer cells are associated with CpG islands. Of the 382 genes whose expression was negatively associated with CpG methylation, 303 (79.3%) were associated with a CpG island as defined by Gardiner-Garden and Frommer [16] (Additional file 1: Table S2 and Figure S7a–b). This is significantly above what would be expected by chance given the frequency of human genes with CpG islands associated. In ten randomly selected groups of 382 genes, the average percentage of genes with associated CpG islands was 66.2% (Fisher exact test *p* < 0.0001).

However, the role of promoter methylation in the transcriptional regulation of genes not associated with CpG islands remains unclear. Here we found that 79 of the 382 genes (20.7%) with significant correlations between mRNA and methylation levels, were not associated with a conventionally defined CpG island. This included PPP1R14D, BST2 and ZNF550, whose expression was induced by 5-aza-2′-deoxycytidine (decitabine) treatment in colon cancer cell lines with low basal expression and high promoter methylation of these genes (Additional file 1: Table S2 and Additional file 1: Fig. S7c–e). These findings suggest that CpG methylation outside conventionally defined CpG islands may also regulate gene expression and contribute to colorectal cancer progression.

### ZBTB18 has tumor suppressor activity in colorectal cancer cells

To gain a better understanding of the biological function of the 382 genes potentially silenced epigenetically during colorectal tumorigenesis, we performed gene set enrichment analysis and identified 15 enriched annotation terms (Fisher’s Exact test, FDR *p* < 0.2; Additional file 1: Table S3). Strikingly, all these largely overlapping categories were related to zinc finger proteins. The largest of these categories (zinc finger region:C2H2-type 4) contained 33 zinc finger domain proteins, including a large proportion of transcriptional regulators (Additional file 1: Table S3).

We postulated that at least some of these epigenetically silenced genes may have tumor suppressor activity in colorectal cancer. As a proof of concept, we investigated the potential tumor suppressive function of the transcriptional repressor ZBTB18 (also known as ZNF238 or RP58; Additional file 1: Figs. S1a, S4a, S6a and Table S2), which belongs to the zinc finger C2H2 family, and whose role in colorectal cancer progression has not been previously addressed.

First, we showed that treatment of four colorectal cancer cell lines with low ZBTB18 expression and high promoter methylation with the demethylating agent decitabine (5-aza-2′-deoxycytidine) resulted in a significant increase of ZBTB18 mRNA expression (Additional file 1: Fig. S8a). Ladakamycin (5-azacytidine), a second demethylating agent used clinically, also upregulated ZBTB18 at the mRNA level, further supporting the notion that ZBTB18 expression is regulated by promoter methylation (Additional file 1: Fig. S8a). Next, we wondered whether ZBTB18 could also be epigenetically regulated by histone modifications. Contrary to DNA methyltransferase inhibitors, drugs interfering with histone acetylation or methylation did not have a significant and consistent impact on ZBTB18 expression (Additional file 1: Fig. S8b).

**Fig. 3:**
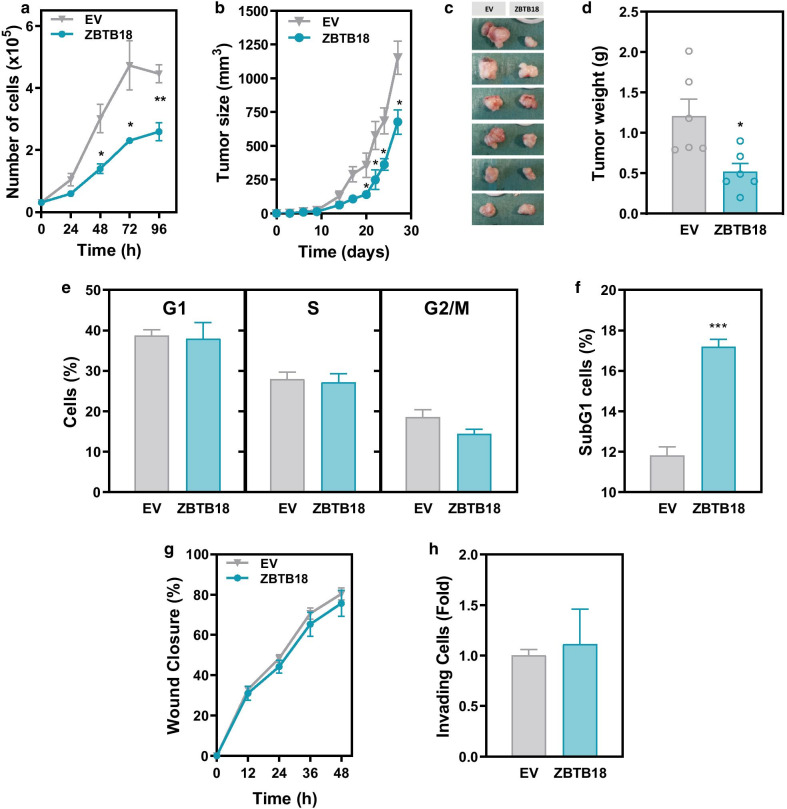
Effects of ZBTB18 overexpression on the growth of colon cancer cells. The effects of ZBTB18 overexpression on the growth of HCT116 cells was assessed by directly counting the number of cells in culture over time (**a**). The growth of HCT116 cells was monitored over time after subcutaneous implantation in immunodeficient NOD/SCID mice (**b**). At the end of the experiment the tumors were dissected out (**c**) and the average weight (± SEM) of the tumors was measured (**d**). Cells in the different phases of the cell cycle (**e**) and SubG1 cells (**f**) were assessed to evaluate the effect of ZBTB18 overexpression in cell growth and apoptosis. Migration (**g**) and invasion (**h**) properties of parental and ZBTB18 overexpressing cells were assessed in wound healing and Matrigel-coated Boyden chamber assays, respectively. Student’s t test *p < 0.05, **p < 0.01 and ***p < 0.001

Next, we engineered two isogenic cell line systems with constitutive or doxycycline-inducible overexpression of bicistronic ZBTB18 and EGFP or control EGFP alone in HCT116 and HT29 cells, respectively (Additional file 1: Fig. S9). FACS analysis demonstrated that > 87% of the cells were EGFP-positive in the HCT116 and HT29 derivative lines (Additional file 1: Fig. S9a and c). Consistently, increased ZBTB18 mRNA expression was confirmed in these derivative cell line systems by qPCR (Additional file 1: Fig. S9b and d). ZBTB18 overexpression in HCT116 and HT29 cells resulted in a significant reduction of their growth in vitro compared to the corresponding EGFP control lines (Fig. 3a and Additional file 1: Fig. S10a). Next, the effects of ZBTB18 overexpression on cell growth were assessed using a subcutaneous xenograft model in NOD/SCID immunodeficient mice. ZBTB18 overexpression resulted in a significant growth reduction of both HCT116 and HT29 cells over the course of four weeks compared to the corresponding EGFP overexpressing control cell lines (Fig. 3b–c and Additional file 1: Fig. S10b–c). Consistently, the weight of the subcutaneous tumors formed by the ZBTB18 overexpressing HCT116 and HT29 cells was significantly lower than in the tumors formed by the corresponding control sublines (Fig. 3d and Additional file 1: Fig. S10d), collectively demonstrating that reintroduction of ZBTB18 into colon cancer cells significantly reduced their growth.

Next, we investigated whether ZBTB18 overexpression caused changes in the cell cycle or the number of apoptotic cells. No significant changes were observed in the distribution of cells along the different phases of the cell cycle in HT29 or HCT116 cells overexpressing ZBTB18 compared to the corresponding control cell lines (Fig. 3e and Additional file 1: Fig. S10e). However, although ZBTB18 overexpression had no effect on the number of apoptotic cells in HT29 cells (Additional file 1: Fig. S10f), increased apoptosis was observed in HCT116 cells overexpressing ZBTB18 compared to control cells (Fig. 3f), likely contributing to the reduced growth observed. We next investigated the role of ZBTB18 on the migration and invasion capacity of colon cancer cells. Ectopic expression of ZBTB18 had no effect on the migration capacity of HT29 or HCT116 cells when assessed in vitro in a wound healing assay. However, ZBTB18 overexpression in HT29 cells significantly reduced their Matrigel invasion capacity compared to control HT29 cells (Fig. 3g–h and Additional file 1: Fig. S10g–h).

### Reduced ZBTB18 expression is associated with poor prognosis of colorectal cancer patients

To further investigate the impact of ZBTB18 silencing in colorectal tumorigenesis, we assessed the possible association between ZBTB18 expression and patient survival in a cohort of 132 cases with locally advanced (Dukes’ C) colorectal cancer. Sections of a tissue microarray containing triplicate samples from these 132 primary tumors, as well as paired normal mucosa samples from a subset of 75 cases and 15 lymph node metastases, were immunostained using a rabbit polyclonal anti-ZBTB18 antibody. The specificity of the antibody was confirmed using formalin-fixed, paraffin-embedded samples of control HCT116 and HT29 (overexpressing EGFP) and the corresponding derivative lines overexpressing ZBTB18 (ZBTB18-IRES-EGFP) grown as subcutaneous xenografts in NOD/SCID immunodeficient mice (Additional file 1: Fig. S11). Moreover, ZBTB18 protein levels assessed with the same antibody in a tissue microarray containing the 30 colorectal cancer cell lines used in this study significantly correlated with ZBTB18 mRNA expression (Additional file 1: Fig. S12), further confirming the specificity of the primary anti-ZBTB18 antibody used.

**Fig. 4:**
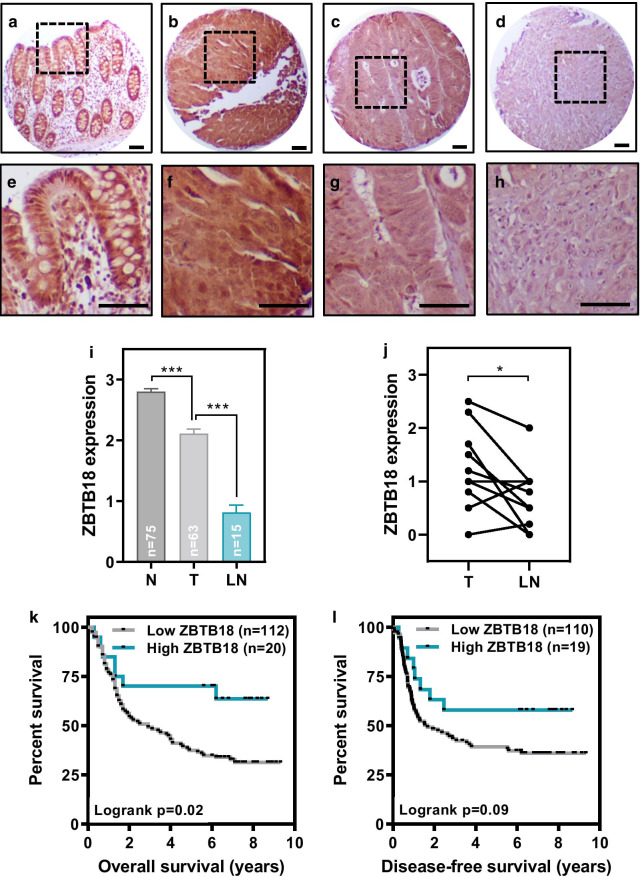
ZBTB18 expression in primary colorectal tumors. Normal colonic epithelial cells had high expression of ZBTB18 (**a**–**b**). Significant variability was observed in the expression of ZBTB18 in colorectal tumors (**c**–**g**). **i** Histogram showing the average (± SEM) expression of ZBTB18 in normal colonic epithelial cells, primary colorectal tumors and lymph node metastases. **j** ZBTB18 expression in 13 primary colorectal tumors and paired lymph node metastasis. **k**–**l** Overall (**k**) and disease-free (**l**) survival as a function of ZBTB18 expression in the tumors from 132 Dukes’ C colorectal cancer patients is shown. N: number of samples. Scale bar: 50 µm. Student’s t test *p < 0.05 and ***p < 0.0001

High ZBTB18 immunostaining was observed in normal colonic epithelial cells (Fig. 4a, b). Significant variability in ZBTB18 expression was observed in primary colorectal tumors, ranging from high levels comparable to the ZBTB18 expression found in normal colonic epithelial cells (Fig. 4c, d), to low or undetectable ZBTB18 expression (Fig. 4e, h). Overall, the average expression observed in primary colorectal tumors was significantly reduced compared to normal colonic epithelial cells (Fig. 4i). Moreover, ZBTB18 protein expression was further reduced in lymph node metastases compared to primary colorectal tumors (Fig. 4i, j). Importantly, an association was observed between low ZBTB18 expression in this cohort of 132 Dukes’ C colorectal cancer patients and shorter overall survival (Fig. 4k). A corresponding trend to shorter disease-free survival was also observed in patients with low ZBTB18 expression in their primary tumors, although it did not reach statistical significance (Fig. 4l). In good agreement, low expression of ZBTB18 mRNA was significantly associated with shorter survival in an independent cohort of 55 Stage III colorectal cancer patients from the TCGA cohort [3, 17] (Additional file 1: Fig. S13). No associations were observed between ZBTB18 expression and other clinicopathological features of the patients in both cohorts (Additional file 1: Tables S4-5).

Collectively, these results demonstrate that ZBTB18 expression is progressively reduced in the transition from normal colonic epithelial cells to primary colorectal carcinoma and lymph node metastasis and that low expression of ZBTB18 in locally advanced primary colorectal tumors is associated with shorter patient survival.

## Discussion

Cytosine methylation in CpG dinucleotides is important during embryonic development and cell differentiation in higher organisms. These methylation marks lead to chromatin condensation and gene silencing and CpG methylation is inherited by the daughter cells after cell division. Aberrant promoter methylation leading to the epigenetic silencing of tumor suppressor genes is an important mechanism leading to the initiation and/or progression of different tumor types, including tumors of the colon and rectum [6]. However, the detailed transcriptional reprograming resulting from aberrant methylation in colorectal tumors has not been thoroughly characterized. The advent of high throughput technologies to interrogate genome-wide levels of mRNA expression and CpG methylation now allows for the systematic identification of genes potentially silenced by DNA methylation.

Here, we analyzed the levels of methylation of 27,578 individual CpGs in a panel of 45 colorectal cancer cell lines and compared them with 223 primary colorectal tumors [3]. It has recently been shown that colorectal cancer cell lines faithfully represent and maintain the genetic diversity of primary tumors [9]. Importantly, we now report that the epigenetically regulated genes in colon cancer cell lines and primary tumors is also highly concordant, indicating that colon cancer cell lines constitute good models to investigate the role of DNA methylation in this disease.

Higher levels of methylation were associated with microsatellite instability, *BRAF* mutations and wild-type *APC* and *TP53* status. This is in good agreement with earlier studies showing that CpG island methylator phenotype (CIMP) positive tumors, a subset of colorectal tumors that has high levels of methylation in CpG islands [18], is associated with microsatellite instability, higher *BRAF* and lower *APC* and *TP53* mutation rates [10, 19, 20]. Hypermethylation of the promoter of *MLH1*, an important component of the DNA mismatch repair system, is common in CIMP + tumors and causes the MSI phenotype observed in most of the CIMP + tumors [3, 10]. This is consistent with our results showing that MSI cell lines and primary tumors have higher levels of methylation. Moreover, in good agreement with our earlier findings showing that MSI colorectal cancer cells proliferate faster than MSS tumor cells [21], and given that MSI tumors have higher levels of methylation, we found here that colon cancer cell lines with higher levels of DNA methylation are associated with faster proliferation rates. Collectively, these results indicate that global promoter methylation in the panel of cell lines derived from colorectal tumors used in this study closely resembles the heterogeneity of DNA methylation observed in primary colorectal tumors.

CpG promoter hypermethylation was observed throughout the genome of colorectal cancer cell lines and primary tumors. However, only the expression of a subset of 382 of the 9,876 genes investigated (3.9%) was negatively associated with the levels of promoter methylation, thus constituting a core set of genes that are potentially regulated by this epigenetic process in colorectal cancer (Additional file 1: Table S2). As expected, these genes included important tumor suppressor genes known to be frequently silenced through promoter methylated in colorectal tumors, such as the cell adhesion protein E-cadherin (*CDH1*) [22], carbonic anhydrase IX (*CA9*) [23–25], the serine/threonine protein kinase *DAPK2* [26], the brush border Myosin Ia (*MYO1A*) [27] and the mismatch repair gene *MLH1* [28]. In addition, these analyses identified other genes with known tumor suppressor activity in colorectal cancer that have not been previously reported to be regulated by promoter methylation in colorectal tumors. These included the chloride channel that is most frequently mutated in patients with cystic fibrosis (*CFTR*) [29, 30] and the negative regulator of Wnt signaling *TCF7* [31].

Methylation of CG dinucleotides in CpG islands and CpG island ‘shores’ is known to regulate gene expression [7]. Although the effect of DNA methylation outside the context of a CpG island has not been as thoroughly investigated, CpG methylation in the promoter of genes without a CpG island has previously been shown to be able to regulate gene expression, often by interfering with the binding of transcriptional regulators [7, 27, 32, 33]. Here, we found that approximately 20% of the 382 genes with expression/methylation correlations are not associated with the presence of a CpG island near their promoter, suggesting that CpG methylation in the promoter of genes not associated with CpG islands may play a significant role in colorectal cancer tumorigenesis.

The list of 382 genes showing a negative association between the levels of gene expression and promoter methylation was significantly enriched in zinc finger proteins (FDR = 9.2 × 10^–6^). Zinc finger proteins constitute a large and diverse family of proteins that contain functional domains that require coordination by at least one zinc ion [34]. Most zinc finger proteins interact with DNA in a sequence-specific manner and regulate gene transcription, although many family members regulate other cellular functions through its capacity to bind to RNA, PAR (poly-ADP-ribose) and other proteins [35]. Some zinc finger proteins have been shown to participate in colorectal tumorigenesis including ZBP89 [36], PRDM1 [37] and ZNF545 [38]. Here, we hypothesized that at least some of the 382 genes showing negative correlations between the levels of expression and methylation are silenced by this epigenetic mechanism and could contribute to the oncogenic process. As a proof of concept, we investigated the role of zinc finger and BTB domain containing 18 (ZBTB18/ZNF238/RP58) in colorectal tumorigenesis. ZBTB18 binds the consensus DNA sequence 5′-(A/C)ACATCTG(G/T)(A/C)-3′, which contains the E box core, and acts by recruiting chromatin remodeling multiprotein complexes such as histone deacetylases, and repressing the expression of downstream genes. ZBTB18 plays a crucial role in supporting myogenesis [39] and brain development [40, 41], although its role in the development of normal intestinal epithelium has not been investigated. The expression of ZBTB18 has been shown to be negatively regulated by methylation of the CpG island in its promoter region [42] and to reduce proliferation as well as to promote apoptosis in brain tumors such as medulloblastoma (MB) and glioblastoma multiforme (GBM) [42, 43]. Moreover, the loss of ZBTB18 has been shown to contribute to the aggressive phenotype of GBM through regulation of poor prognosis-associated signatures [42]. However, the role of this zinc finger protein in colorectal tumorigenesis has not been investigated. Although ZBTB18 is not frequently mutated in colorectal cancer (0.3–2.5% in large exome sequencing studies [3, 44, 45]), here we found that the expression of ZBTB18 was downregulated by promoter methylation in colorectal cancer cells and that its overexpression reduced their growth both in vitro and in subcutaneous xenograft mouse models. Therefore, strategies aimed at rescuing ZBTB18 expression or downregulating some of the downstream ZBTB18 targets genes may have therapeutic potential in colorectal cancer patients.

The expression of several zinc finger proteins, such as ZEB1 [46], ZFX [47] and PRDM1 [37], has been reported to be associated with the survival of colorectal cancer patients. Moreover, an association has been reported between high levels of ZBTB18 promoter methylation and shorter progression-free survival of patients with glioblastomas [42]. Here, a validated anti-ZBTB18 antibody was used to assess the expression of this zinc finger protein in normal colonic samples as well as locally advanced primary colorectal tumors and lymph node metastases. While high ZBTB18 expression was observed in normal colonic epithelial cells, a significant reduction of ZBTB18 immunostaining was frequently observed in primary colorectal tumors. Reduced ZBTB18 expression in the primary tumors was associated with shorter patient survival and the average expression of ZBTB18 was further reduced in lymph node metastases compared to primary colorectal tumors. These findings are consistent with the tumor suppressor activity observed when ZBTB18 was overexpressed in colon cancer cells, and indicate that the levels of expression of this transcriptional repressor could be useful to identify a subset of locally advanced colorectal cancer patients with poor prognosis that are good candidates to receive a more aggressive form of treatment and/or closer follow up.

In summary, we used here transcriptomic and methylomic genome-wide analysis of colorectal cancer cell lines and primary colorectal tumors to identify a subset of 382 genes with a negative association between expression and methylation levels and that likely include epigenetically regulated genes driving the oncogenic process. Zinc finger proteins were significantly enriched in this list of 382 genes with expression/methylation associations, and we demonstrated the tumor suppressor activity of the zinc finger transcriptional repressor ZBTB18 in colon cancer cells. Consistently, reduced ZBTB18 expression in locally advanced primary colorectal tumors was associated with shorter patient survival. The role of other genes in the core set of 382 genes with expression/methylation associations on colorectal tumorigenesis remains to be characterized in future investigations.

## Methods

### Cell lines

A total of 45 colorectal cancer cell lines were used (see Additional file 1). All lines were maintained in MEM (Life Technologies, Carlsbad, CA) supplemented with 10% fetal bovine serum, 1 × antibiotic/antimycotic, 1 × MEM Non-Essential Amino Acids Solution, and 10 mM HEPES buffer solution (all from Life Technologies, Carlsbad, CA) at 37 °C and 5% CO_2_. All lines were tested to be negative for mycoplasma contamination (PCR Mycoplasma Detection Set, Takara) and possible cell line cross-contamination was ruled out by clustering analysis of genome-wide mRNA expression and promoter methylation data at the time of these experiments.

### ZBTB18 overexpression in colon cancer cells

HCT116 cells were transfected with pIRES2-EGFP or pZBTB18-IRES-EGFP with Lipofectamine 2000 (Thermo Fisher Scientific, Waltham, MA). For tetracycline-inducible overexpression of ZBTB18, HT29 cells were transduced with pINDUCER20-EGFP or pINDUCER20-ZBTB18-IRES-EGFP as previously described [48] and further described in the ‘Supplementary Materials’.

### Determination of cell growth

3 × 10^4^ HT29, HCT116 or derivative cells were seeded in triplicate wells of seven 24-well plates and allowed to adhere overnight. Cells were trypsinized and stained with trypan blue at the indicated times. Growth curves presented were plotted with Prism (GraphPad Software).

### Quantification of mRNA expression

Microarray analysis was used for genome-wide expression profiling of 30 colorectal cell lines (Human Genome U133 Plus 2.0 Array; Affymetrix, Santa Clara, CA; ArrayExpress E-MTAB-2971) and 223 primary colorectal tumors (Agilent 244 K Custom Gene Expression G4502A-07–1/2/3) [3]. Relative mRNA levels of selected genes were assessed by Real-Time PCR using SYBR Green Master Mix (Applied Biosystems, Branchburg, NJ) as described before [21]. Primers sequence is available in Additional file 1: Table S1.

### Assessment of DNA methylation

Methylation at the single-nucleotide resolution level for a total of 27,578 CpG sites was determined using HumanMethylation27 Beadchip (Illumina, San Diego, CA) for a panel of 45 colorectal cancer cell lines (See Supplementary Methods; ArrayExpress MTAB-7867). Promoter methylation data for a cohort of 223 primary colorectal tumors (Illumina HumanMethylation27 Beadchip) was available from The Cancer Genome Atlas (TCGA) [3]. In this study, tumors from all stages (American Joint Committee on Cancer staging system) and histologic grade were collected from newly diagnosed patients with colon or rectum adenocarcinomas undergoing surgical resection. None of the patients received neoadjuvant chemotherapy or radiotherapy. For bisulfite sequencing analysis, genomic DNA was bisulfite treated with EZ DNA Methylation-Gold Kit (Zymo Research, Irvine, CA) following the manufacturer’s instructions and then PCR-amplified. Primers used are listed in Additional file 1: Table S1.

### Associations between mRNA expression and promoter methylation levels

Using the gene symbol as a common identifier, we found that expression data were available for a total of 11,359 (81.92%) of the 14,475 promoters interrogated in the HumanMethylation27 arrays. Significant correlations between gene expression and promoter methylation were identified using Spearman correlations (False Discovery Rate (FDR) < 0.2). Genes showing a nonlinear negative association between expression and methylation levels (i.e., ‘L-shaped’ in the scattered plots; see Additional file 1: Fig. S5) were identified using the ad hoc generated tool available at http://cinna.upc.edu:3838/alex/Lheuristic/ (additional details described in [49]).

### Gene set enrichment analysis

To investigate whether there was an enrichment in the number of genes with significant correlation between expression and methylation levels that belonged to different categories of functionally related genes, we used the Database for Annotation, Visualization and Integrated Discovery (DAVID) v6.7 [50].

### Subcutaneous xenograft mouse model

Subcutaneous tumors were established in NOD/SCID mice by injection HCT116 or HT29 derivative cells resuspended in 100 μl sterile PBS. All animals bearing tumors from HCT116 derivative cells received 1 mg/ml doxycycline (Sigma) in the drinking water and tumor size followed over time. At the experimental end point, all animals were euthanized, tumors were surgically removed, and the tumor weight was determined. Tumor specimens were formalin-fixed and paraffin-embedded for histological analysis. All experiments were carried out under observance of the protocol approved by the Ethical Committee for Animal Experimentation from the Vall d’Hebron University Hospital Institute of Research (Barcelona, Spain).

### Determination of cell cycle and apoptosis

Cell cycle and hypodiploid (subG1) cells were quantified by flow cytometry after propidium iodide staining. Full procedure is detailed in the ‘Supplementary Materials’.

### Cell migration and invasion assays

To investigate changes in the motility and invasion of HCT116 and HT29 cells upon ZBTB18 upregulation, a wound healing and a Matrigel Boyden chamber assay were used, respectively [48]. Further details are described in Additional file 1: ‘Supplementary Materials’.

### Tissue microarrays analysis

TMAs from colorectal cancer cell lines (Supplementary Methods) and colorectal cancer patients were used [51]. Briefly, 30 colorectal cancer cell lines, 75 samples of normal colon tissues, 132 primary adenocarcinomas from colon and rectum and 15 lymph node metastases from Dukes’ C Finnish colorectal cancer patients were formalin-fixed, paraffin-embedded and arrayed in triplicate.

### Immunohistochemistry

Tumor samples excised from mouse xenograft experiments and tissue microarrays (TMAs) were immunostained for ZBTB18 protein detection using a rabbit polyclonal anti-ZBTB18 antibody at 1:1000 dilution (Biorbyt, Cambridge, UK; cat# orb357631).

## Supplementary Information

**Additional file 1: Supplementary Figures: Supplementary Figure S1**. CpG methylation levels assessed by HumanMethylation27 beadChips (Illumina) and direct bisulfite sequencing. **Supplementary Figure S2**. CpG methylation in primary colorectal tumors. **Supplementary Figure S3**. Associations between the average levels of methylation and molecular features of primary colorectal tumors and cell lines. **Supplementary Figure S4**. Representative examples of genes showing significant correlations between mRNA and methylation levels. **Supplementary Figure S5**. representative genes showing a non-linear negative association between mRNA expression and methylation levels. **Supplementary Figure S6**. Validation of the differences in gene expression observed by microarray analysis. **Supplementary Figure S7**. Effects of decitabine treatment on the expression of genes with promoter methylation. **Supplementary Figure S8**. Effects of DNMT (Decitabine and Ladakamycin), HDAC (Vorinostat) KDM (OG-L002) and KMT (Tazemetostat) inhibitors on the expression of ZBTB18. **Supplementary Figure S9**. Validation of ZBTB18 overexpression in colon cancer cell line systems. **Supplementary Figure S10**. Effects of ZBTB18 overexpression on the growth of HT29 colon cancer cells. **Supplementary Figure S11**. ZBTB18 antibody validation. **Supplementary Figure S12**. ZBTB18 protein expression in colorectal cell lines. **Supplementary Figure S13**. Survival of Stage III colorectal cancer patients as a function of ZBTB18 mRNA expression. **Supplementary Tables: Supplementary Table S1**. PCR primer s used in this study. **Supplementary Table S2**. Details of the 382 gene s showing significant correlation between expression and methilation levels in 30 colorectal cell lines and 223 primary tumors. **Supplementary Table S3**. Group enrichment analysis: Functional group enrichment analysis carried out with DAVID (https://doi.org/10.1186/gb-2003-4-9-r60). **Supplementary Table S4** Clinicopathological features of the 132 Dukes C colorectal cancer patients in this study as a function of ZBTB18 protein expression as determined by immunohistochemistry. **Supplementary Table S5** Clinicopathological features of the 55 colorectal Stage III cancer patients from the TCGA as a function of ZBTB 18 mRNA expression as determined by microarray analysis (Agilent). **Supplementary Methods**.

## Data Availability

Microarray and methylation data of the cell lines are available in ArrayExpress E-MTAB-2971 and MTAB-7867.

## Abbreviations

MSI: Microsatellite instable
MSS: Microsatellite stable
CIMP: CpG island methylator phenotype
MEM: Minimum essential medium
PCR: Polymerase chain reaction
TCGA: The Cancer Genome Atlas
NOD/SCID: Nonobese diabetic/severe combined immunodeficiency
FDR: False discovery rate
EGFP: Enhanced green fluorescent protein
