# Supplementary material for: Identification of ZBTB18 as a novel colorectal tumor suppressor gene through genome-wide promoter hypermethylation analysis
Source: Clin Epigenetics. 2021 Apr 23;13:88. doi: 10.1186/s13148-021-01070-0 (PMC8063439; doi:10.1186/s13148-021-01070-0)
Supplement: Supplementary file 1 — Additional file 1: Supplementary Figures: Supplementary Figure S1. CpG methylation levels assessed by HumanMethylation27 beadChips (Illumina) and direct bisulfite sequencing. Supplementary Figure S2. CpG methylation in primary colorectal tumors. Supplementary Figure S3. Associations between the average levels of methylation and molecular features of primary colorectal tumors and cell lines. Supplementary Figure S4. Representative examples of genes showing significant correlations between mRNA and methylation levels. Supplementary Figure S5. representative genes showing a non-linear negative association between mRNA expression and methylation levels. Supplementary Figure S6. Validation of the differences in gene expression observed by microarray analysis. Supplementary Figure S7. Effects of decitabine treatment on the expression of genes with promoter methylation. Supplementary Figure S8. Effects of DNMT (Decitabine and Ladakamycin), HDAC (Vorinostat) KDM (OG-L002) and KMT (Tazemetostat) inhibitors on the expression of ZBTB18. Supplementary Figure S9. Validation of ZBTB18 overexpression in colon cancer cell line systems. Supplementary Figure S10. Effects of ZBTB18 overexpression on the growth of HT29 colon cancer cells. Supplementary Figure S11. ZBTB18 antibody validation. Supplementary Figure S12. ZBTB18 protein expression in colorectal cell lines. Supplementary Figure S13. Survival of Stage III colorectal cancer patients as a function of ZBTB18 mRNA expression. Supplementary Tables: Supplementary Table S1. PCR primer s used in this study. Supplementary Table S2. Details of the 382 gene s showing significant correlation between expression and methilation levels in 30 colorectal cell lines and 223 primary tumors. Supplementary Table S3. Group enrichment analysis: Functional group enrichment analysis carried out with DAVID (https://doi.org/10.1186/gb-2003-4-9-r60). Supplementary Table S4 Clinicopathological features of the 132 Dukes C colorectal cancer patients in thi [file 13148_2021_1070_MOESM1_ESM.pdf]

Identification of ZBTB18 as a novel colorectal tumor suppressor gene through genome-wide promoter hypermethylation analysis

Sarah Bazzocco[1*], Higinio Dopeso[1*], Águeda Martínez-Barriocanal[1,8*], Estefanía Anguita[1], Rocío Nieto[1], Jing Li[1], Elia García-Vidal[1], Valentina Maggio[1], Paulo Rodrigues[1], Priscila Guimarães de Marcondes[1], Simo Schwartz Jr[2,3], Lauri A. Aaltonen[4], Alex Sánchez[5], John M. Mariadason[6,7], Diego Arango[1,8]

SUPPLEMENTARY FIGURES S1-S13

Supplementary Figure S1

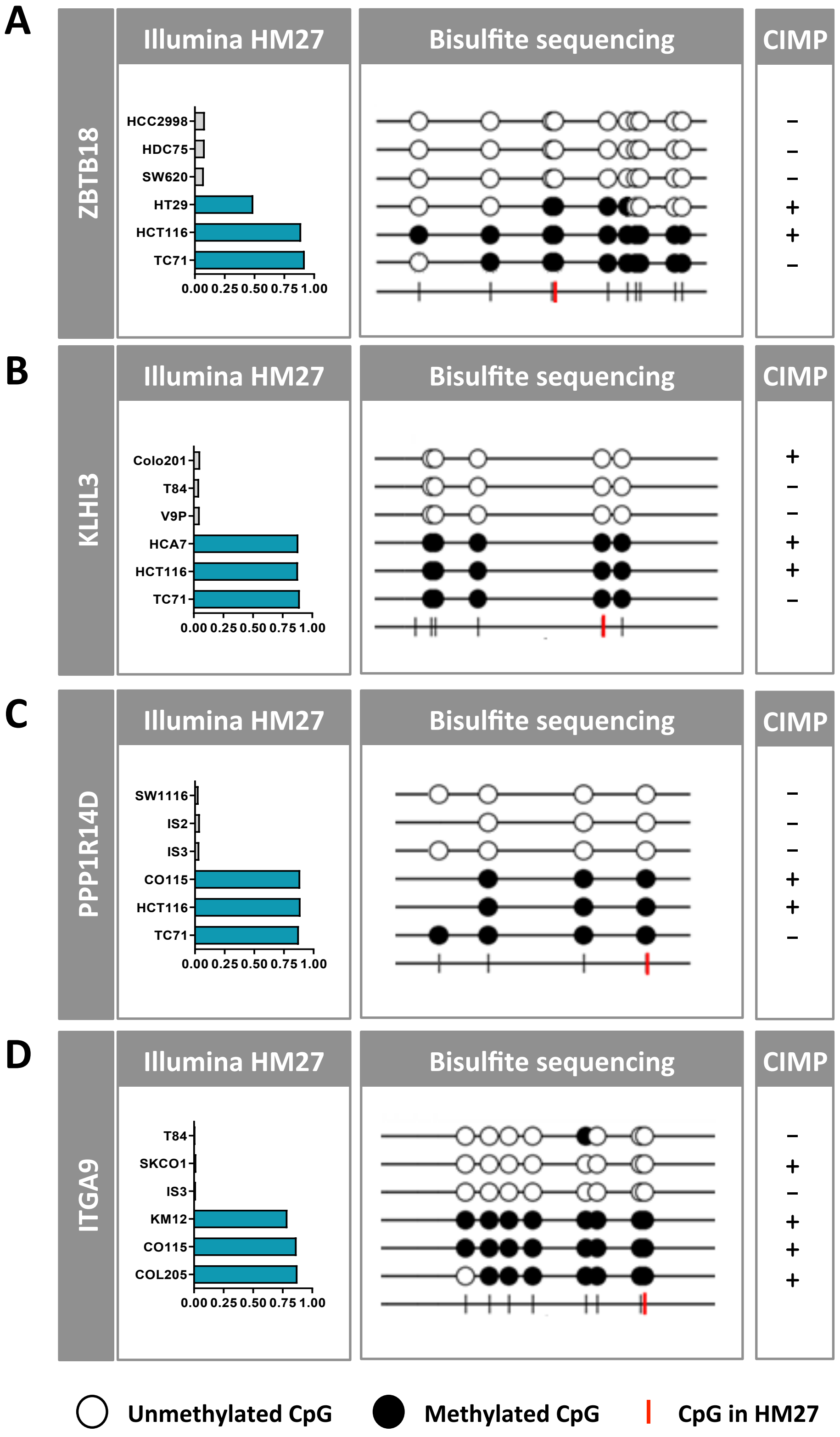

Supplementary Figure S1: CpG methylation levels assessed by HumanMethylation27 beadChips (Illumina) and direct bisulfite sequencing. The levels of promoter methylation observed in the genes ZBTB18 (**A**), KLHL3 (**B**), PPP1R14D (**C**) and ITGA9 (**D**) by Illumina's HumanMethylation27 BeadChip (left panels) and bisulfite sequencing (middle panels; white circle: unmethylated CpG; back circles: methylated CpG; red lines: CpG interrogated in the HumanMethylation27 BeadChip) are shown for the colorectal cancer cell lines indicated. CIMP status for the cell lines assayed is indicated (right panels).

Supplementary Figure S2

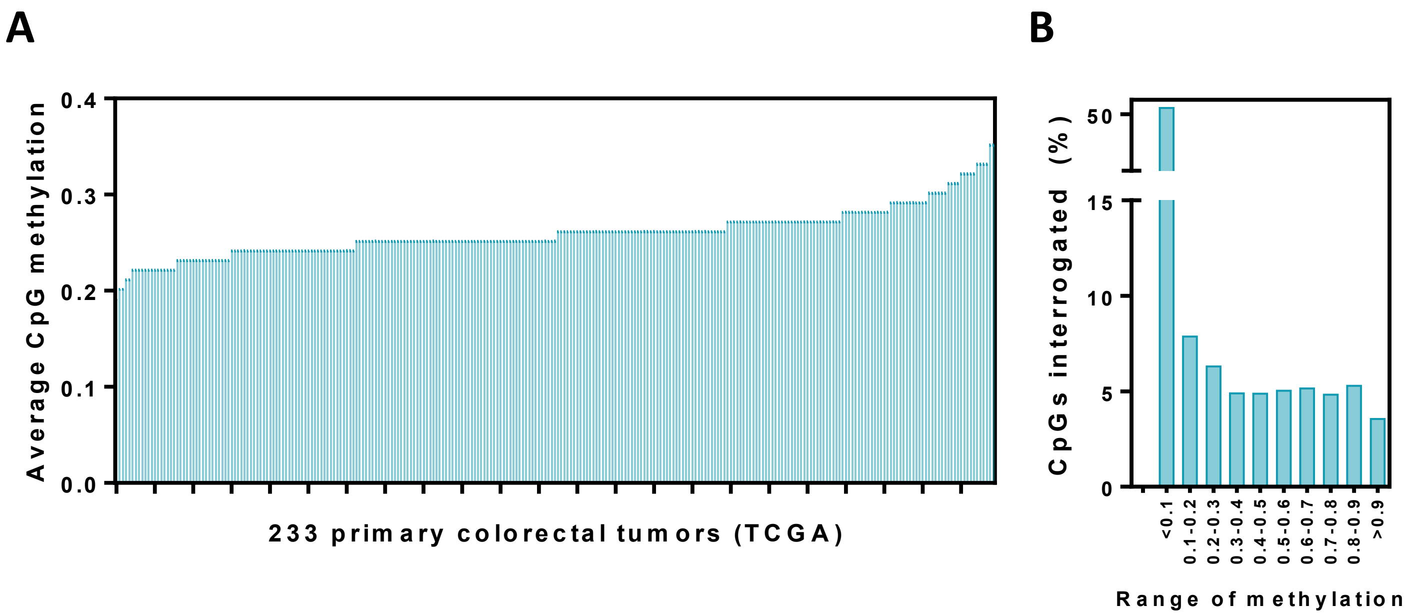

Supplementary Figure S2: CpG methylation in primary colorectal tumors. A) A cohort of 223 primary colorectal tumors form the TCGA (DOI: 10.1038/nature11252) was used. The average methylation of all the 27,578 CpGs interrogated, representing 14,495 genes, was variable and ranged from 0.27 to 0.35 with an overall average across all 223 primary tumors of 0.26±0.02 (mean±SD). **B**) Histogram showing the percentage of the CpG dinucleotides interrogated that have average methylation levels across the 223 primary tumors in the indicated intervals.

Supplementary Figure S3

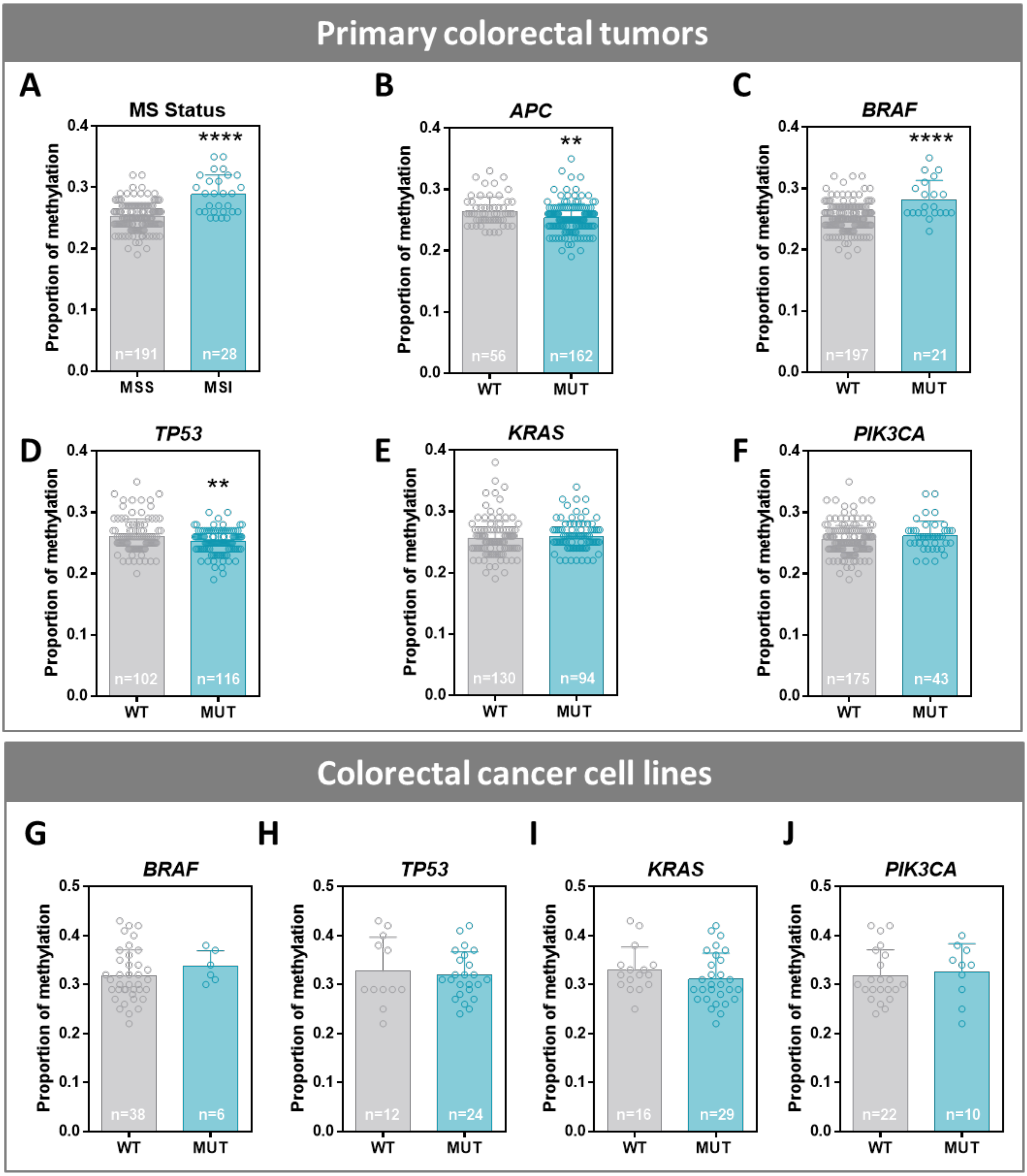

Supplementary Figure S3: Associations between the average levels of methylation and molecular features of primary colorectal tumors and cell lines. (**A-F**) Using a cohort of 223 primary colorectal tumors from the TCGA (DOI: 10.1038/nature11252), higher levels of CpG methylation were found to be associated with microsatellite instability (**A**), wild type *APC* status (**B**), mutant *BRAF* status (**C**) and wild type *TP53* status (**D**). No associations were found between methylation levels and mutations in *KRAS* (**E**) and *PIK3CA* (**F**). In a panel of 45 colorectal cancer cell lines, no associations were found between the average levels of methylation and mutations in *BRAF* (**G**), *TP53* (**H**), *KRAS* (**I**) and *PIK3CA* (**J**). N: number of samples.

Supplementary Figure S4

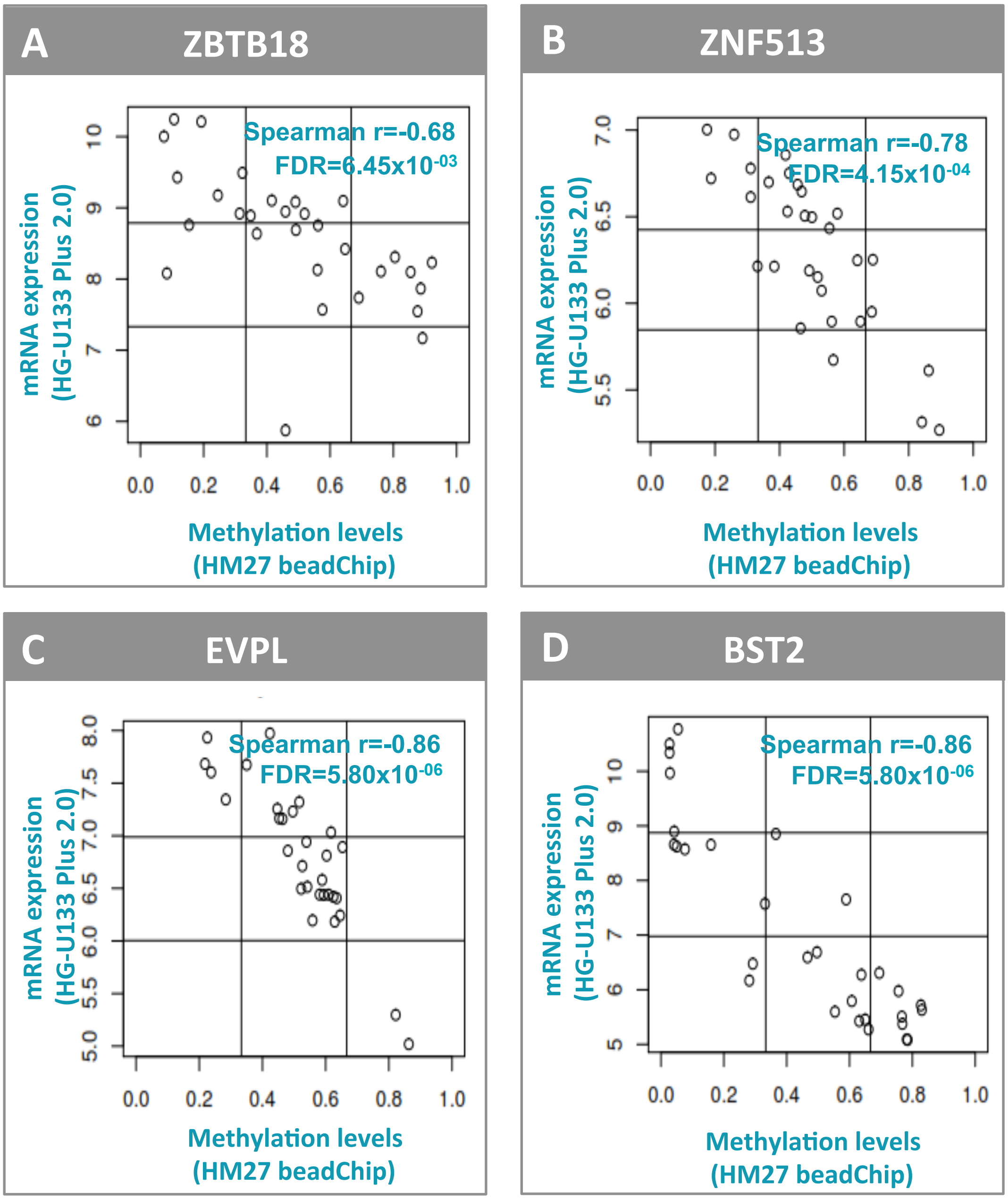

Supplementary Figure S4: Representative examples of genes showing significant correlations between mRNA and methylation levels. Panels **A-D** show examples of genes with significant negative Spearmen's correlations between mRNA expression and promoter methylation levels. The Spearman's correlation coefficients and the False Discovery Rate (FDR) q-values are shown.

Supplementary Figure S5

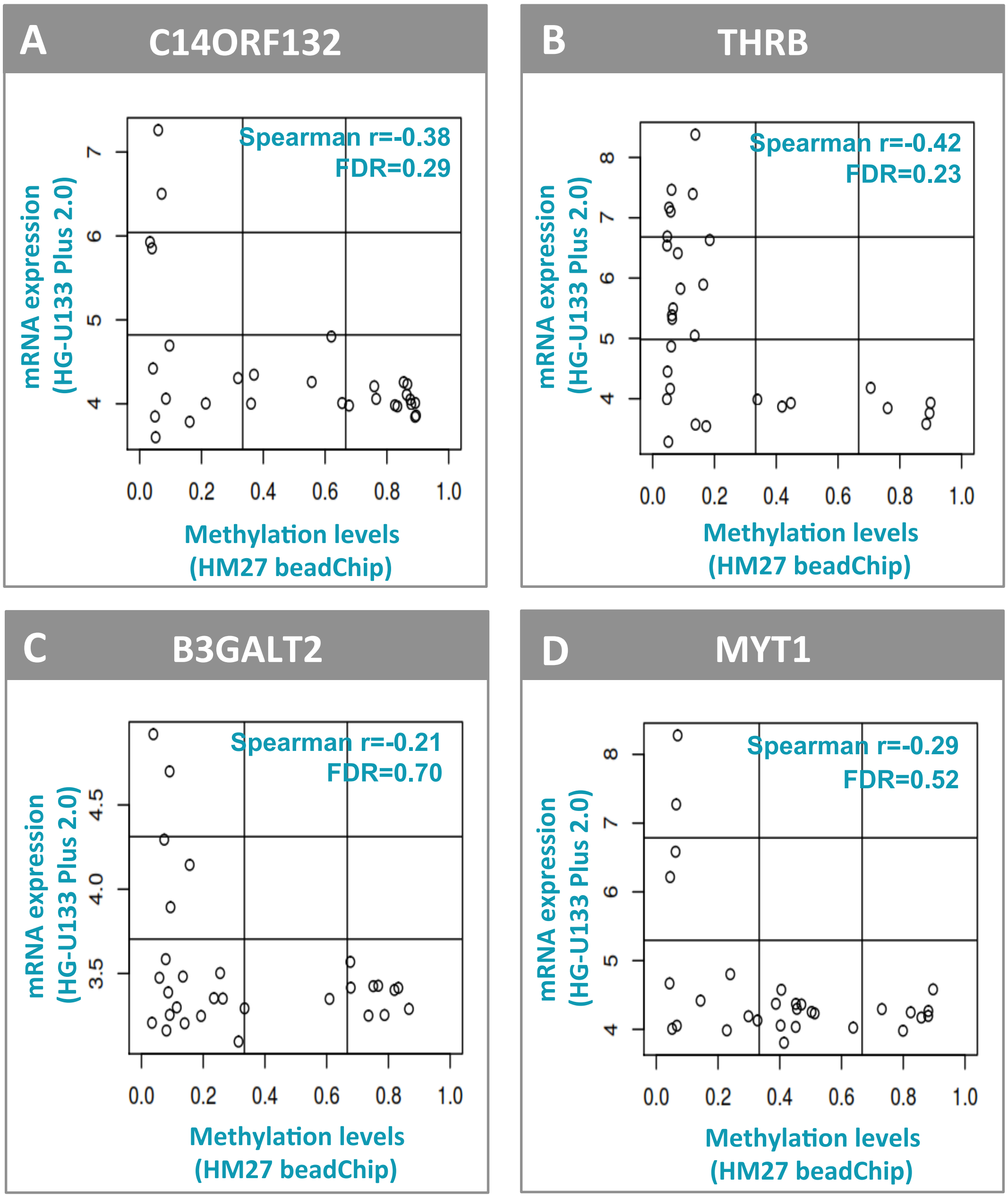

Supplementary Figure S5: representative genes showing a non-linear negative association between mRNA expression and methylation levels. Panels **A-D** are representative examples of genes with no significant Spearmen's correlations between mRNA and promoter methylation levels, but show the expected 'L-shaped' profile characteristic of genes whose expression is regulated by promoter methylation in this type of plots. The Spearman's correlation coefficients and the False Discovery Rate (FDR) q-values are shown.

Supplementary Figure S6

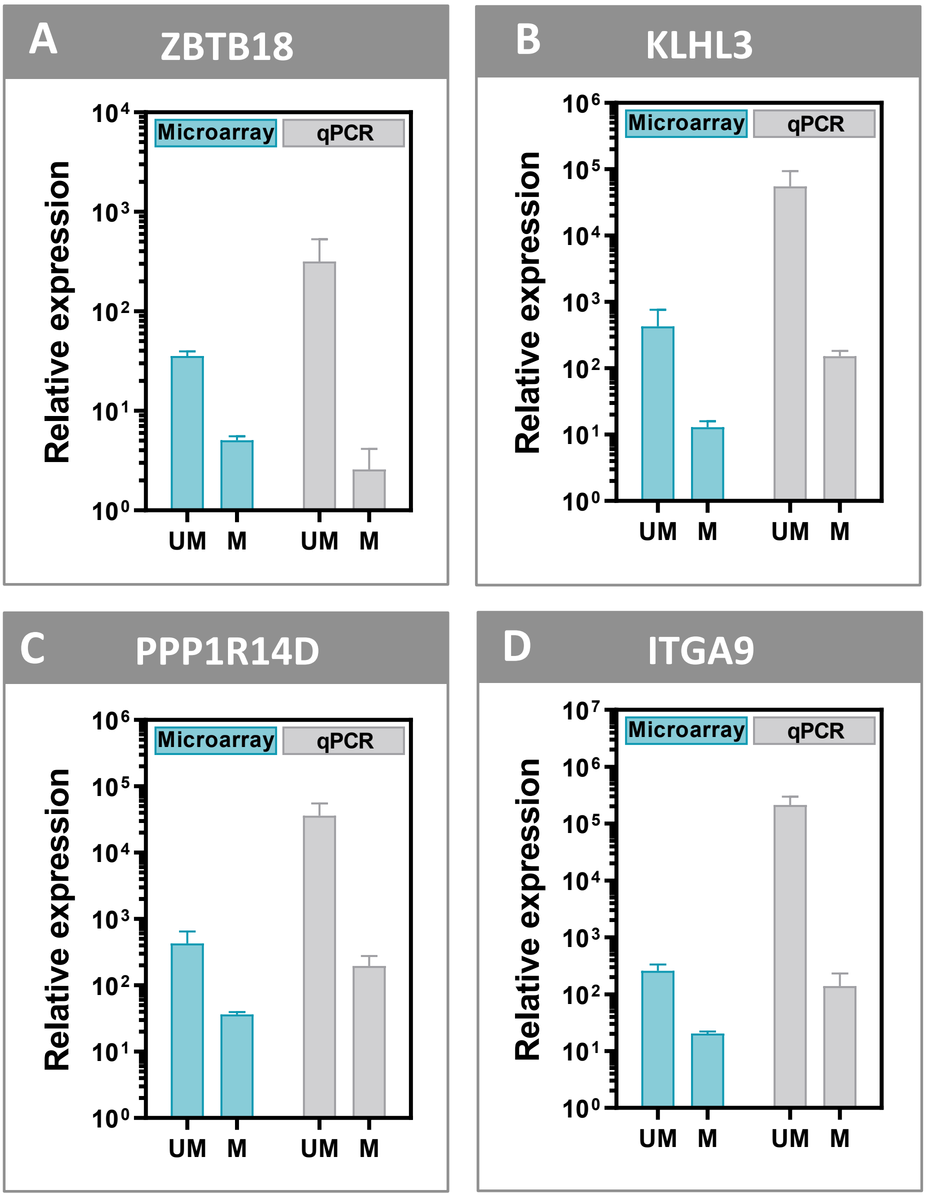

Supplementary Figure S6: Validation of the differences in gene expression observed by microarray analysis. The expression differences observed by microarray analysis for the genes ZBTB18 (**A**), KLHL3 (**B**), PPP1R14D (**C**) and ITGA9 (**D**) was confirmed by quantitative Real-Time RT-PCR in three cell lines with low methylation and high expression (UM) and three lines with high methylation and low expression (M) for each of the genes. Each column in this histogram represents the average expression in three M/UM cell lines for each gene.

Supplementary Figure S7

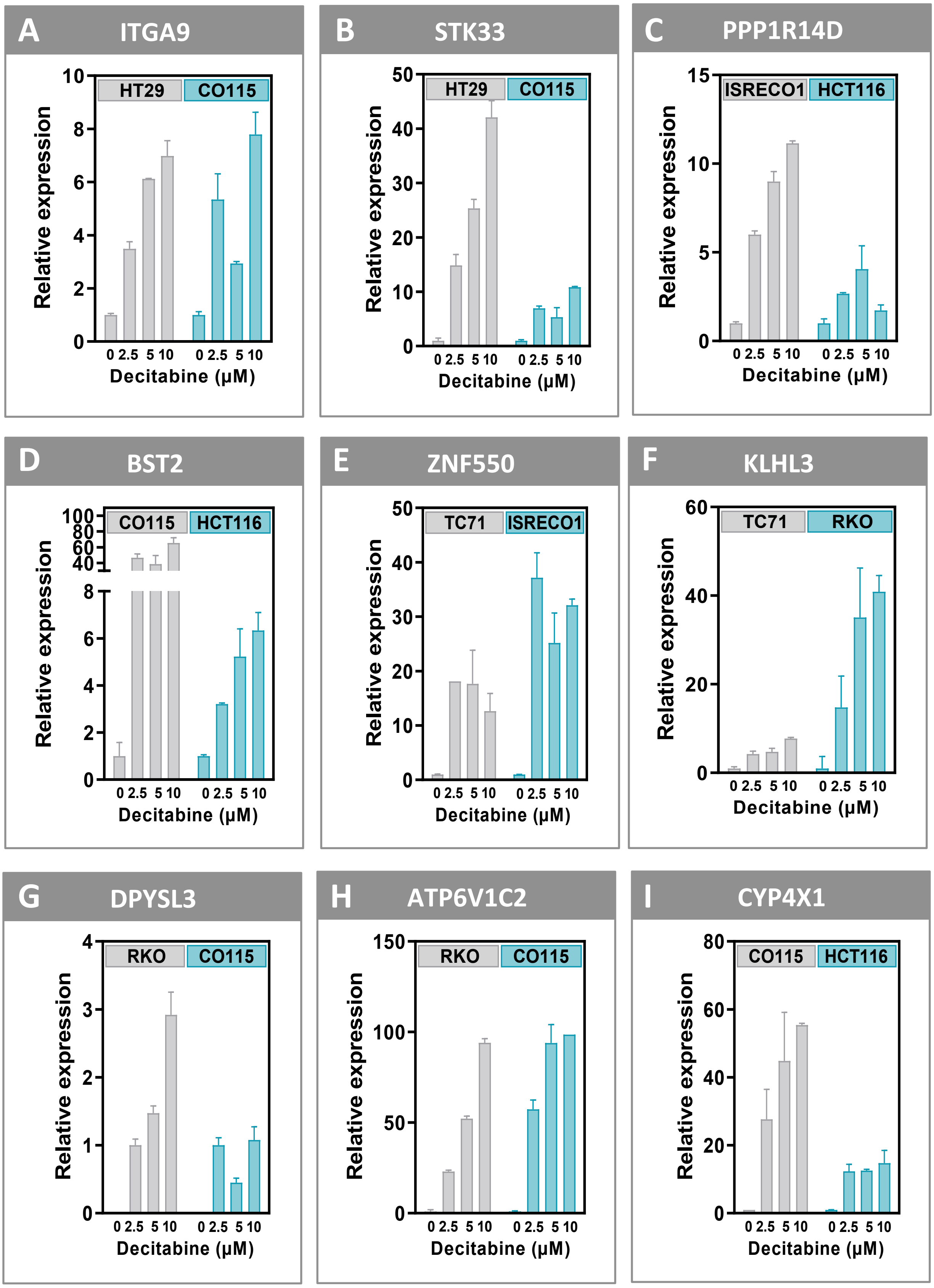

Supplementary Figure S7: Effects of decitabine treatment on the expression of genes with promoter methylation. Cell lines with low levels of expression and high levels of methylation of the indicated genes were treated with increasing concentration of the DNA methyltransferase inhibitor decitabine (5-Aza-2′-deoxycytidine) for 72 hours. Treatment with the inhibitor resulted in increased expression of ITGA9 (**A**), STK33 (**B**), PPP1R14D (**C**), BST2 (**D**), ZNF550 (**E**), KLHL3 (**F**), DPYSL3 (**G**), ATP6V1C2 (**H**), and CYP4X1 (**I**) measured by Real-Time RT-PCR.

Supplementary Figure S8

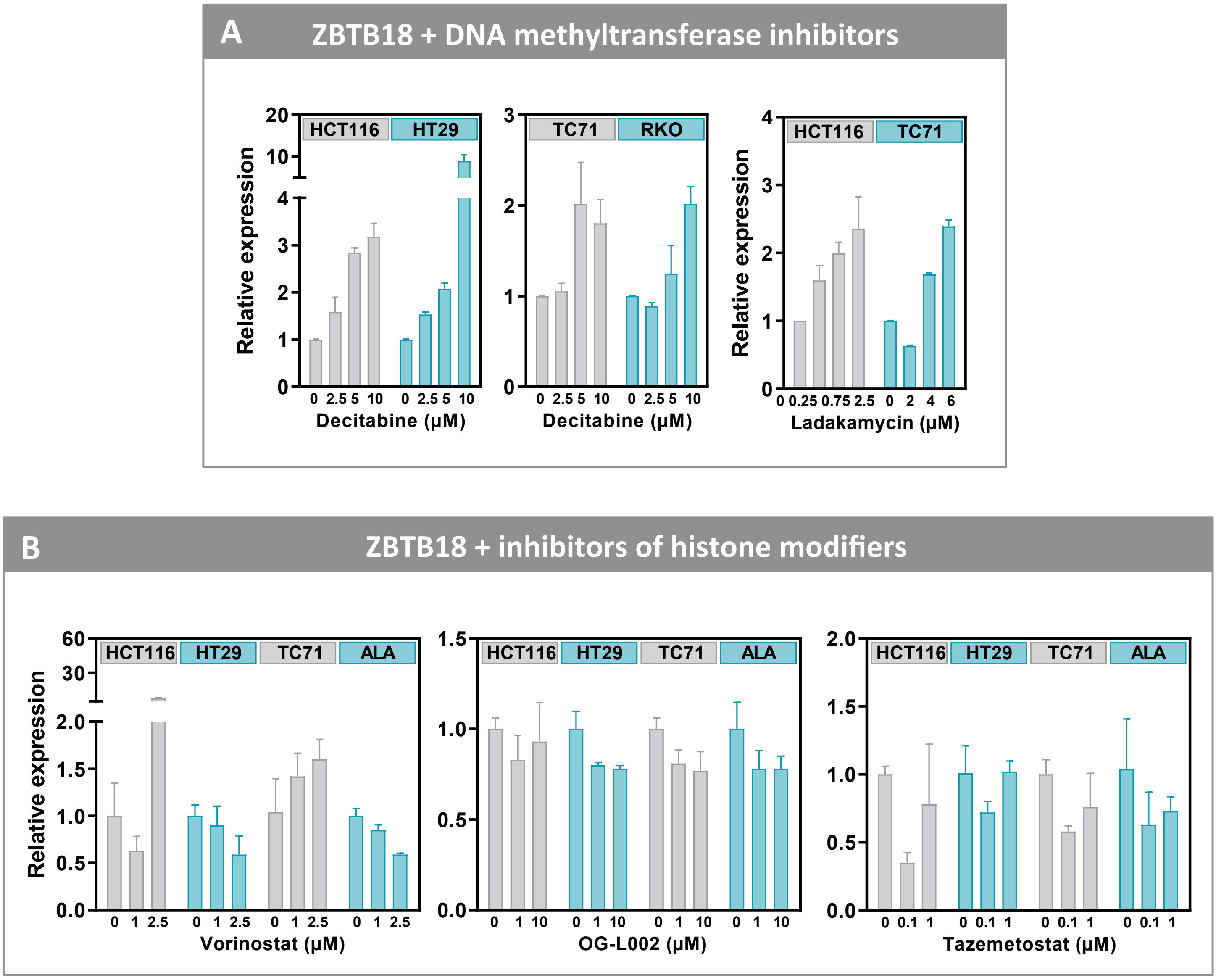

Supplementary Figure S8: Effects of DNMT (Decitabine and Ladakamycin), HDAC (Vorinostat) KDM (OG-L002) and KMT (Tazemetostat) inhibitors on the expression of ZBTB18. A) Colorectal cancer cell lines with low levels of expression and high levels of methylation of ZBTB18 were treated with increasing concentrations of the DNA methyltransferase inhibitors decitabine and ladakamycin for 72h. Treatment with both drugs resulted in increased expression of ZBTB18 measured by Real-Time RT-PCR. **B**) Colorectal cancer cell lines were treated with increasing concentrations of the histone deacetylase inhibitor vorinostat, the lysine demethylase inhibitor OG-L002 and the lysine methyltransferase inhibitor tazemetostat for 72h. ZBTB18 transcript levels were not modified consistently upon measurement by Real-Time PCR.

Supplementary Figure S9

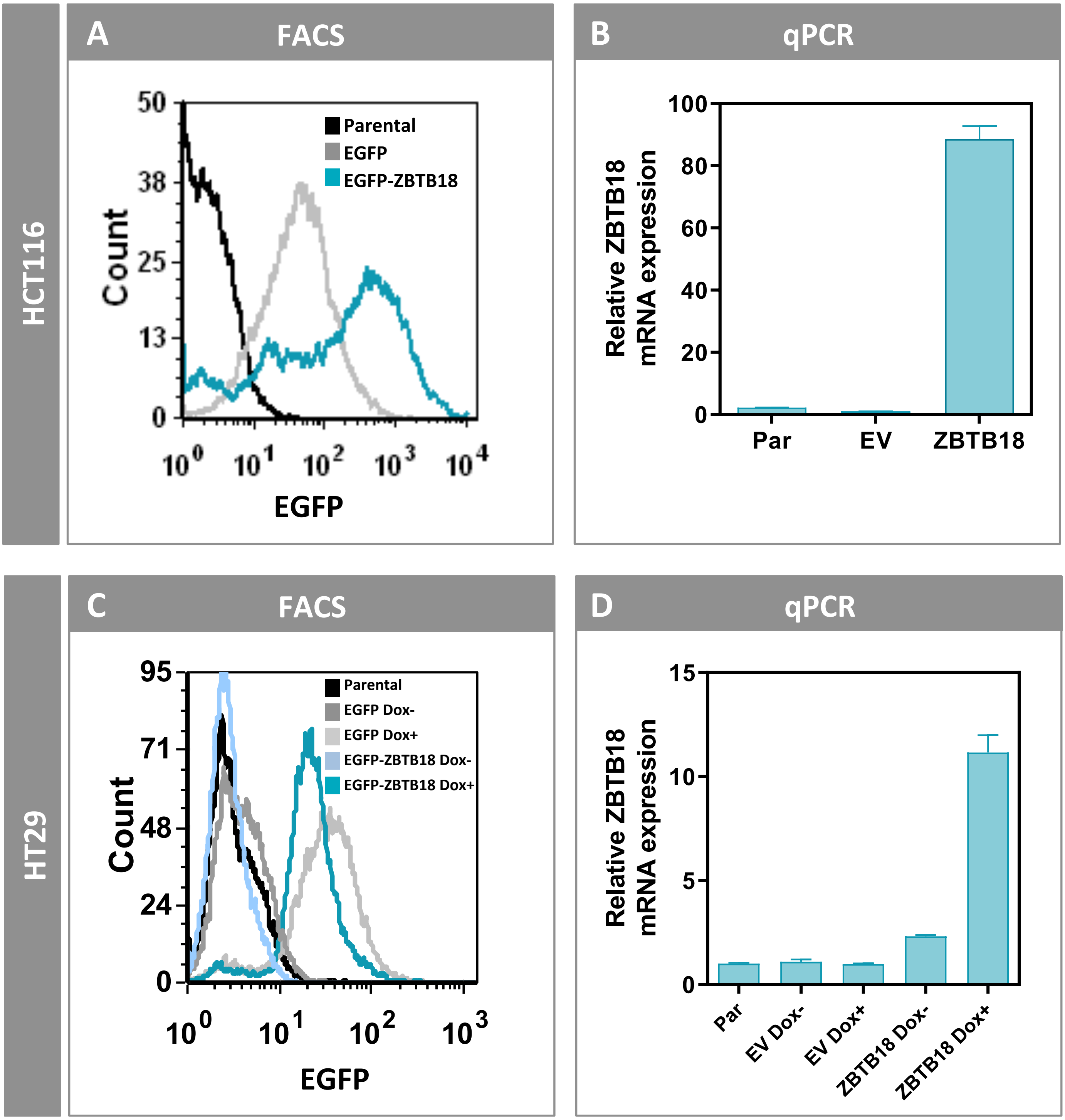

Supplementary Figure S9: Validation of ZBTB18 overexpression in colon cancer cell line systems. EGFP-ZBTB18 was stably overexpressed constitutively into HCT116 and conditionally (Tet-ON system) into HT29 cells using lentiviral vectors. EGFP expression was assessed by FACS in HCT116 (**A**) and HT29 (**C**). ZBTB18 overexpression was confirmed by qPCR in HCT116 and HT29 (**B** and **D**). The mean of two independent determinations (±SEM) is shown in **B** and **D**.

Supplementary Figure S10

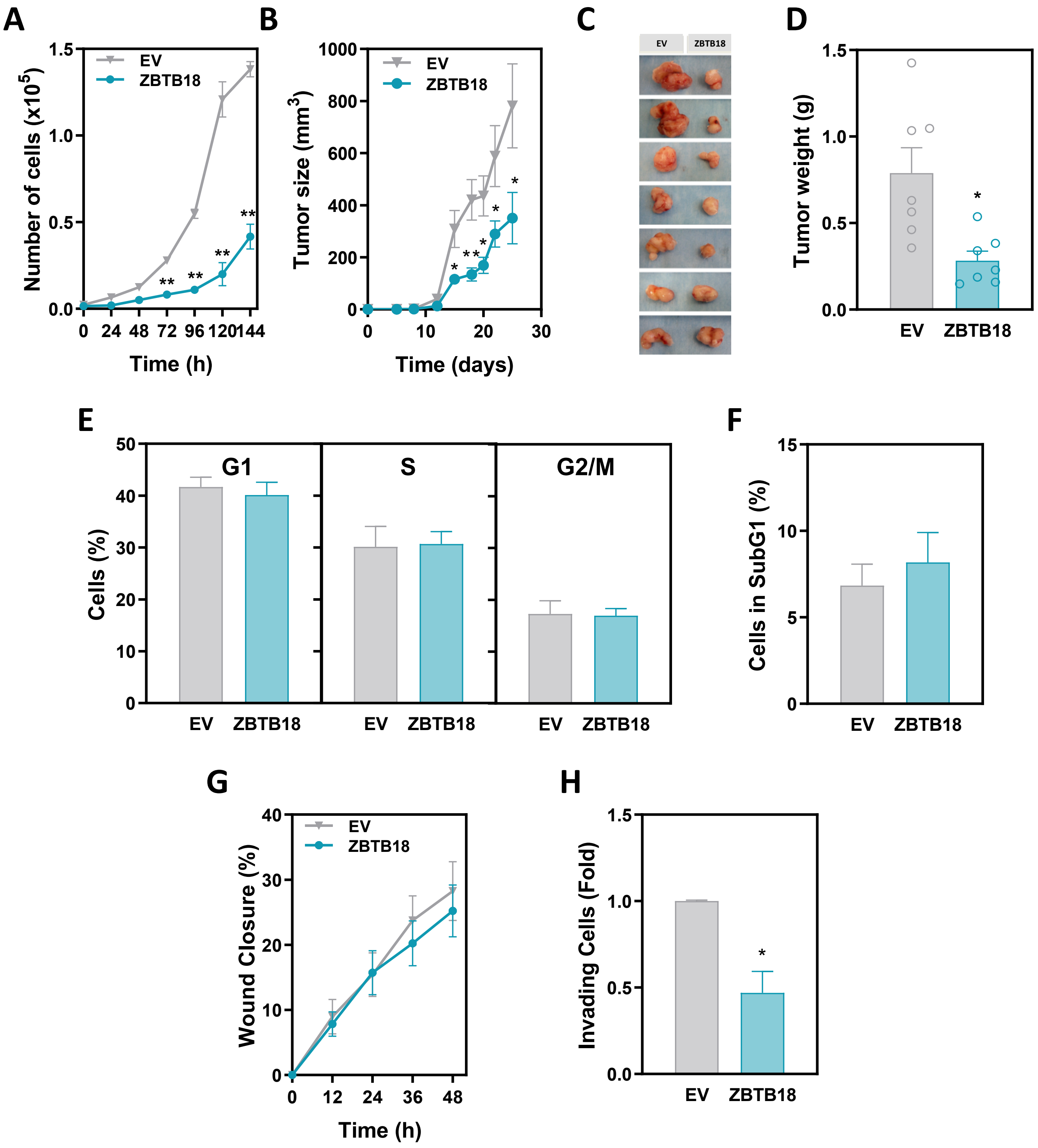

Supplementary Figure S10: Effects of ZBTB18 overexpression on the growth of HT29 colon cancer cells. The effects of ZBTB18 overexpression on the growth of HT29 cells was assessed by directly counting the number of cells in culture over time (**A**). The growth of HCT116 cells was monitored over time after subcutaneous implantation in immunodeficient NOD/SCID mice (**B**). At the end of the experiment the tumors were dissected out (**C**) and the average weight (±SEM) of the tumors was measured (**D**). Cells in the different phases of the cell cycle (**E**) and SubG1 cells (**F**) were assessed to evaluate the effect of ZBTB18 overexpression in cell growth and apoptosis. Migration (**G**) and invasion (**H**) properties of parental and ZBTB18 overexpressing cells were interrogated in wound healing and matrigel-coated boyden chamber assays, respectively. Student's T-test *p<0.05 and **p<0.01.

Supplementary Figure S11

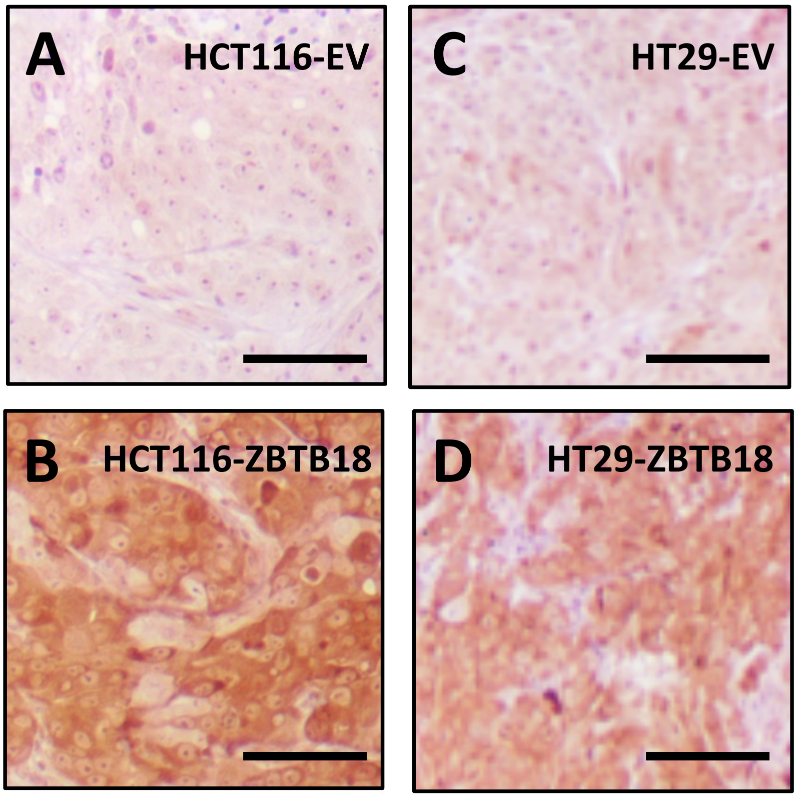

Supplementary Figure S11: ZBTB18 antibody validation. Detection of ZBTB18 on formalin-fixed, paraffin-embedded samples was validated using a rabbit polyclonal antibody on xenografts tumors from control (EV) (**A**, **C**) and ZBTB18 overexpressing (**B**, **D**) HCT116 and HT29 cells. Scale bar: 50µm.

Supplementary Figure S12

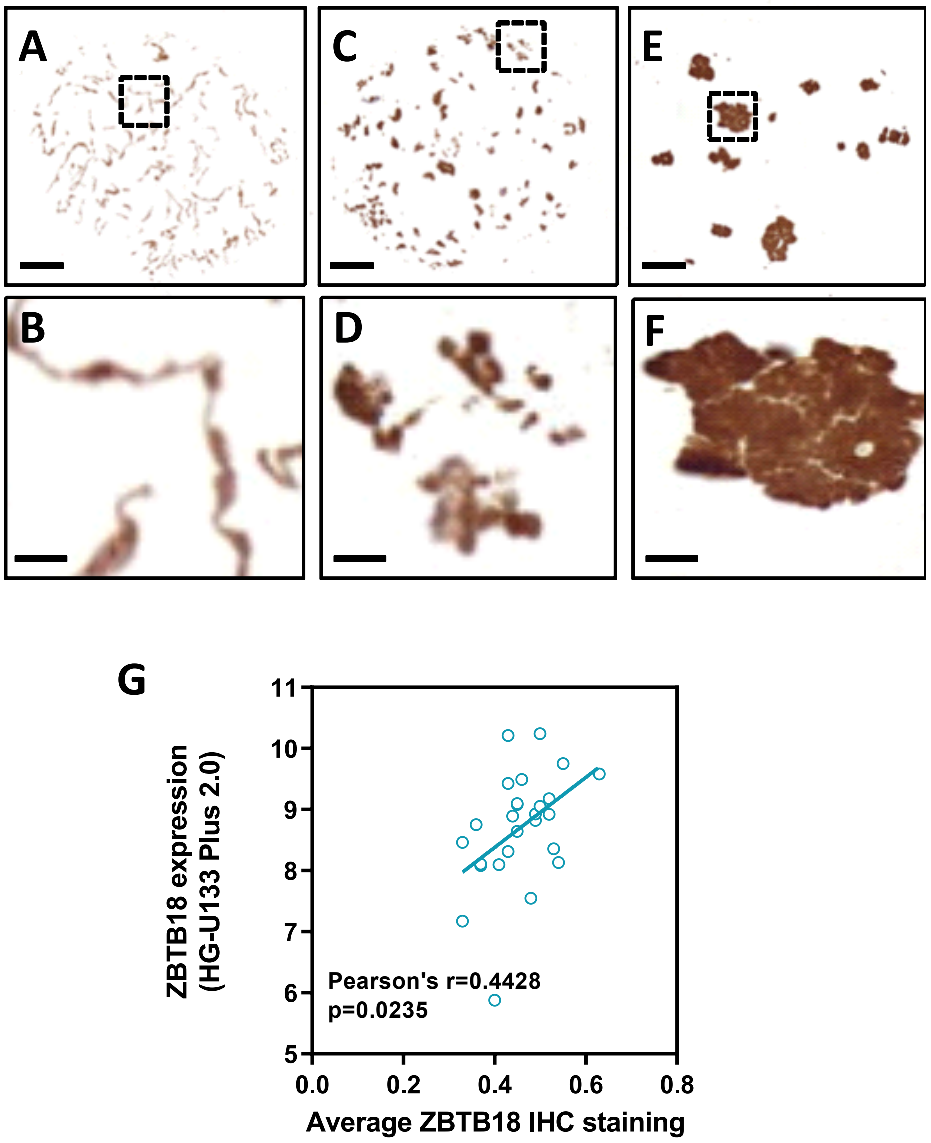

Supplementary Figure S12: ZBTB18 protein expression in colorectal cell lines. Detection of ZBTB18 on formalin-fixed, paraffin-embedded cell lines in a tissue microarray. Significant variability was observed in the expression of ZBTB18 in colorectal cell lines (**A-F**). ZBTB18 protein levels correlated with ZBTB18 transcript levels in the HG-U133 Plus2.0 microarray (**G**). Scale bar: 200µm (upper panels), 25µm (lower panels).

Supplementary Figure S13

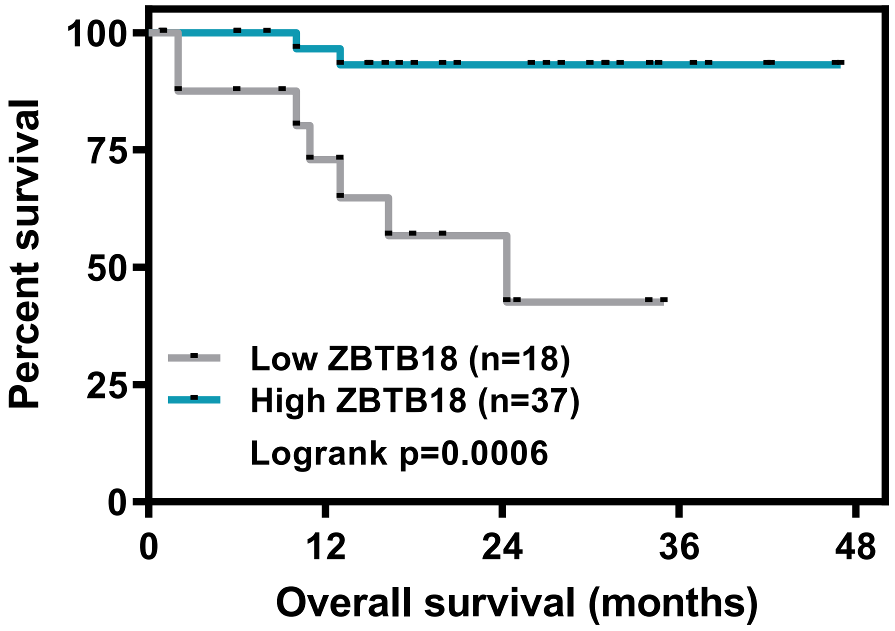

Supplementary Figure S13: Survival of Stage III colorectal cancer patients as a function of ZBTB18 mRNA expression. The levels of ZBTB18 mRNA expression in a cohort of 55 Stage III colorectal cancer patients from the TCGA (DOI: 10.1038/nature11252) were assessed by microarray analysis (Agilent). Overall survival is shown in patients with high or low ZBTB18 expression.

Identification of ZBTB18 as a novel colorectal tumor suppressor gene through genome-wide promoter hypermethylation analysis

Sarah Bazzocco[1*], Higinio Dopeso[1*], Águeda Martínez-Barriocanal[1,8*], Estefanía Anguita[1], Rocío Nieto[1], Jing Li[1], Elia García-Vidal[1], Valentina Maggio[1], Paulo Rodrigues[1], Priscila Guimarães de Marcondes[1], Simo Schwartz Jr[2,3], Lauri A. Aaltonen[4], Alex Sánchez[5], John M. Mariadason[6,7], Diego Arango[1,8]

SUPPLEMENTARY TABLES S1-S5

Supplementary Table S1: PCR primers used in this study.

Primers name	Application[1]	Location	Sequence 5'-3'
STK33-qPCR-F	qPCR	Exon 2-4	GGAGCTGCTATTGAGGAAATC
STK33-qPCR-R	qPCR	Exon 2-4	CTTCACAGCAGAGCTTCCAG
BST2-qPCR-F	qPCR	Exon 1-4	CACACTGTGATGGCCCTAATG
BST2-qPCR-R	qPCR	Exon 1-4	GTCCGCGATTCTCACGCTT
ZNF550-qPCR-F	qPCR	Exon 2-4	TTTCACTAGGGCATCGGGTT
ZNF550-qPCR-R	qPCR	Exon 2-4	GGGTAAGTGGTTGGCTCTCT
KLHL3-qPCR-F	qPCR	Exon 10-12	AGTACTGGCCTAGCATCGGT
KLHL3-qPCR-R	qPCR	Exon 10-12	CGGGAAGCTCCATCATAAC
PPP1R14D-qPCR-F	qPCR	Exon 3-5	AGACTCAGCTGGAGGCCAT
PPP1R14D-qPCR-R	qPCR	Exon 3-5	CAGTGCTGAGGCTGCTAAAG
ITGA9-qPCR-F	qPCR	Exon 27-28	GTTGGTGGGAATCCTCATCT
ITGA9-qPCR-R	qPCR	Exon 27-28	AAAGAAGCCCATCTTCCAGA
ZBTB18-qPCR-F	qPCR	Exon 1-2	AGCAGGACTCAGAGGAAAGG
ZBTB18-qPCR-R	qPCR	Exon 1-2	CCAGAACAGTGCAGTCACAA
DPYSL3-qPCR-F	qPCR		CTCCTCTGGTTGTCATCTGC
DPYSL3-qPCR-R	qPCR		CAGGTCTGCCATCTTCCTC
ATP6V1C2-qPCR-F	qPCR	Exon 12-13	CCGTGCTCAGGTATGGACTA
ATP6V1C2-qPCR-R	qPCR	Exon 12-13	TCTTAAACGCTTGGTGGATG
CYP4X1-qPCR-F	qPCR	Exon 8-9	CTGAACCCTGAGCATCAAGA
CYP4X1-qPCR-R	qPCR	Exon 8-9	CAGCTGGTCCCAAGTGATAG
18S-F	qPCR	-	AGTCCCTGCCCTTTGTACACA
18S-R	qPCR	-	GATCCGAGGGCCTCACTAAAC
18S-Probe	qPCR	-	[6FAM]-CGCCCGTCGCTACTACCGATTGG-[TAM]
ITGA9-BS-F	BSS	cg13882267	GGGATTTGAGGATTTGTATTTTTT
ITGA9-BS-R	BSS	cg13882267	CCTCTACTCCTTCACCCAATTATAA
PPP1R14D-BS-F	BSS	cg23382741	AGGTTAGGTTGATAGTAGTTTATATT
PPP1R14D-BS-R	BSS	cg23382741	CCTCTATATCCCACCTTCCTAAAAC
KLHL3-BS-F	BSS	cg13847070	AAGTTGGAAAGGTGGTAGTGTATTT
KLHL3-BS-R	BSS	cg13847070	CCAACAAACCAATAAAAAATCTAATC
ZBTB18-BST-F1a	BSS	cg02497700	TTTTTTATTTTATTGGGTAAATGGG
ZBTB18-BST-R1a	BSS	cg02497700	CCCAACCCTAATAATAACCACTTC
ZBTB18-BST-F1b	BSS	cg02497700	GGACTCATTAAACTGTCACTCACC
ZBTB18-BST-R1b	BSS	cg02497700	AGGGGTAGAAAAGGAACTGGG
ZBTB18-BST-F2	BSS	cg23829949	TATATAGATAGGGAGTTAGTGTGT
ZBTB18-BST-R2	BSS	cg23829949	TATACTCAATCTAATCTCTTACTAC
BamHI-Kozak-ZBTB18-F	Cloning	-	TCGCAGGGATCCGCCGCCATGGAGTTTCCAGACCATAGTAGAC
ZBTB18-BamHI-R	Cloning	-	TCACGCGGATCCTTATTTCCAAAGTTCTTGAGAGCTATC
AttB1-Kozak-ZBTB18-F	Cloning	-	GGGGACAAGTTTGTACAAAAAAGCAGGCTTCGCCACCATGGAGTTTCCAGACCATA
AttB1-IRES2-F	Cloning	-	GGGGACAAGTTTGTACAAAAAAGCAGGCTTCGCCCCTCTCCCTCCCC
AttB2-EGFP-R	Cloning	-	GGGGACCACTTTGTACAAGAAAGCTGGGTCTTACTTGTACAGCTCGTCCATGCC

[1]qPCR: quantitative RT-PCR; BSS: primers used for PCR amplification for bisulfite sequnceing; Cloning: PCR amplification for plasmid

Supplementary Table S2. Details of the 382 genes showing significant correlation between expression and methylation levels in 30 colorectal cell lines and 223 primary colorectal tumors (TCGA).

Gene ID	Name	UGCluster	Spearman r (cell lines)	Spearman p (cell lines)	FDR adjusted (cell lines)	L-shape (cell lines)	Spearman r (primay tumors)	Spearman p (primay tumors)	FDR adjusted (primay tumors)	L-shape (primay tumors)	CpG islands (promoter;intragenic)[1]
BST2	bone marrow stromal cell antigen 2	Hs.118110	-0,87	6,90E-10	5,80E-06	FALSE	-0,82	0,00E+00	0,00E+00	FALSE	0;0
RIPK3	receptor-interacting serine-threonine kinase 3	Hs.268551	-0,86	1,36E-09	5,80E-06	FALSE	-0,20	2,78E-03	1,53E-02	FALSE	0;1
EVPL	envoplakin	Hs.500635	-0,86	1,53E-09	5,80E-06	FALSE	-0,15	2,69E-02	8,99E-02	FALSE	0;0
PYCARD	PYD and CARD domain containing	Hs.499094	-0,85	3,63E-09	9,57E-06	FALSE	-0,35	5,75E-08	1,17E-06	FALSE	1;1
ZNF420	zinc finger protein 420	Hs.444992	-0,85	4,21E-09	9,57E-06	TRUE	-0,14	3,95E-02	1,21E-01	FALSE	1;1
HSPA1A	heat shock 70kDa protein 1A	Hs.274402	-0,81	4,47E-08	7,26E-05	FALSE	-0,68	0,00E+00	0,00E+00	FALSE	2;1
FADS1	fatty acid desaturase 1	Hs.503546	-0,79	1,83E-07	2,59E-04	FALSE	-0,13	5,42E-02	1,51E-01	FALSE	1;3
ZNF141	zinc finger protein 141	Hs.654355	-0,78	3,09E-07	3,91E-04	FALSE	-0,42	6,89E-11	2,25E-09	FALSE	1;1
LY75	lymphocyte antigen 75	Hs.153563	-0,78	4,09E-07	4,16E-04	FALSE	-0,23	6,00E-04	4,37E-03	FALSE	1;1
ZNF550	zinc finger protein 550	Hs.180257	-0,78	4,64E-07	4,16E-04	FALSE	-0,57	0,00E+00	0,00E+00	FALSE	0;0
ZNF513	zinc finger protein 513	Hs.515872	-0,78	4,64E-07	4,16E-04	FALSE	-0,16	1,61E-02	6,07E-02	FALSE	1;2
TGFBI	transforming growth factor, beta-induced, 68kDa	Hs.369397	-0,77	5,38E-07	4,16E-04	FALSE	-0,14	3,76E-02	1,16E-01	FALSE	1;1
GPX2	glutathione peroxidase 2	Hs.2704	-0,77	6,08E-07	4,16E-04	FALSE	-0,28	2,58E-05	2,99E-04	FALSE	0;0
RIBC2	RIB43A domain with coiled-coils 2	Hs.475110	-0,77	6,08E-07	4,16E-04	FALSE	-0,43	1,84E-11	6,57E-10	FALSE	1;1
PRAP1	proline-rich acidic protein 1	Hs.15951	-0,76	8,90E-07	5,61E-04	TRUE	-0,37	1,58E-08	3,58E-07	FALSE	0;0
RUSC2	RUN and SH3 domain containing 2	Hs.493796	-0,76	9,99E-07	5,97E-04	FALSE	-0,45	1,69E-12	7,08E-11	FALSE	1;1
TACSTD2	tumor-associated calcium signal transducer 2	Hs.23582	-0,76	1,12E-06	6,37E-04	FALSE	-0,13	4,72E-02	1,37E-01	FALSE	1;1
LGALS1	lectin, galactoside-binding, soluble, 1	Hs.445351	-0,76	1,38E-06	7,44E-04	FALSE	-0,15	2,40E-02	8,29E-02	FALSE	0;1
ZNF14	zinc finger protein 14	Hs.659932	-0,75	1,72E-06	8,67E-04	FALSE	-0,41	1,62E-10	5,14E-09	FALSE	2;1
PPP1R14D	protein phosphatase 1, regulatory (inhibitor) subunit 14D	Hs.192927	-0,75	1,76E-06	8,67E-04	FALSE	-0,40	4,95E-10	1,43E-08	FALSE	0;0
FXYD3	FXYD domain containing ion transport regulator 3	Hs.301350	-0,75	2,09E-06	9,90E-04	FALSE	-0,14	4,06E-02	1,23E-01	FALSE	0;0
HMGCS2	3-hydroxy-3-methylglutaryl-CoA synthase 2 (mitochondrial)	Hs.59889	-0,75	2,18E-06	9,92E-04	FALSE	-0,65	0,00E+00	0,00E+00	FALSE	0;0
NGEF	neuronal guanine nucleotide exchange factor	Hs.97316	-0,74	2,94E-06	1,24E-03	FALSE	-0,31	2,22E-06	3,34E-05	FALSE	1;3
ZNF502	zinc finger protein 502	Hs.224843	-0,74	3,62E-06	1,47E-03	FALSE	-0,52	0,00E+00	0,00E+00	FALSE	1;1
MRPS21	mitochondrial ribosomal protein S21	Hs.405880	-0,73	3,84E-06	1,51E-03	FALSE	-0,73	0,00E+00	0,00E+00	FALSE	0;1
RAB32	RAB32, member RAS oncogene family	Hs.287714	-0,73	4,17E-06	1,53E-03	FALSE	-0,45	2,53E-12	1,02E-10	FALSE	1;1
CA9	carbonic anhydrase IX	Hs.63287	-0,73	4,52E-06	1,56E-03	TRUE	-0,33	3,56E-07	6,19E-06	FALSE	0;1
S100P	S100 calcium binding protein P	Hs.2962	-0,73	5,74E-06	1,86E-03	FALSE	-0,25	1,99E-04	1,74E-03	FALSE	0;0
CARD14	caspase recruitment domain family, member 14	Hs.675480	-0,71	1,05E-05	2,60E-03	FALSE	-0,35	5,64E-08	1,16E-06	FALSE	0;4
TNFRSF1B	tumor necrosis factor receptor superfamily, member 1B	Hs.256278	-0,71	1,05E-05	2,60E-03	FALSE	-0,28	2,16E-05	2,56E-04	FALSE	1;2
LYZ	lysozyme	Hs.524579	-0,71	1,07E-05	2,60E-03	FALSE	-0,29	1,52E-05	1,88E-04	TRUE	0;0
SPAG16	sperm associated antigen 16	Hs.743365	-0,71	1,07E-05	2,60E-03	FALSE	-0,51	4,44E-16	2,88E-14	FALSE	1;2
ZNF71	zinc finger protein 71 (Cos26)		-0,71	1,07E-05	2,60E-03	FALSE	-0,47	1,45E-13	6,98E-12	FALSE	1;3
ZNF256	zinc finger protein 256	Hs.596242	-0,71	1,20E-05	2,84E-03	FALSE	-0,74	0,00E+00	0,00E+00	FALSE	2;1
LMCD1	LIM and cysteine-rich domains 1	Hs.475353	-0,70	1,69E-05	3,82E-03	FALSE	-0,30	6,62E-06	8,99E-05	FALSE	1;1
ASCL2	achaete-scute family bHLH transcription factor 2	Hs.152475	-0,70	1,84E-05	4,02E-03	FALSE	-0,22	7,60E-04	5,31E-03	FALSE	1;1
IQGAP2	IQ motif containing GTPase activating protein 2	Hs.291030	-0,69	2,08E-05	4,38E-03	FALSE	-0,31	2,14E-06	3,22E-05	FALSE	1;4
EPS8L3	EPS8-like 3	Hs.485352	-0,69	2,15E-05	4,45E-03	FALSE	-0,31	3,33E-06	4,84E-05	FALSE	0;0
IRX2	iroquois homeobox protein 2		-0,69	2,43E-05	4,84E-03	FALSE	-0,68	0,00E+00	0,00E+00	FALSE	1;1
ESPN	espin	Hs.744222	-0,69	2,55E-05	5,00E-03	FALSE	-0,34	1,44E-07	2,73E-06	FALSE	2;6
SGK2	serum/glucocorticoid regulated kinase 2	Hs.300863	-0,69	2,87E-05	5,44E-03	FALSE	-0,53	0,00E+00	0,00E+00	FALSE	0;0
ALDH3A1	aldehyde dehydrogenase 3 family, member A1	Hs.531682	-0,69	2,92E-05	5,44E-03	FALSE	-0,12	6,71E-02	1,77E-01	FALSE	0;1
GIPC2	GIPC PDZ domain containing family, member 2	Hs.603818	-0,68	3,62E-05	6,32E-03	FALSE	-0,23	7,13E-04	5,04E-03	FALSE	1;2
FUT3	fucosyltransferase 3 (galactoside 3(4)-L-fucosyltransferase, Lewis blood group)	Hs.169238	-0,68	3,86E-05	6,45E-03	FALSE	-0,28	2,23E-05	2,63E-04	FALSE	0;1
TSPAN15	tetraspanin 15	Hs.499941	-0,68	3,86E-05	6,45E-03	FALSE	-0,14	3,35E-02	1,07E-01	FALSE	1;1
ZBTB18	zinc finger and BTB domain containing 18	Hs.69997	-0,68	3,86E-05	6,45E-03	FALSE	-0,45	2,22E-12	9,04E-11	FALSE	1;3
DYRK1B	dual-specificity tyrosine-(Y)-phosphorylation regulated kinase 1B	Hs.130988	-0,67	5,31E-05	7,94E-03	FALSE	-0,35	1,16E-07	2,25E-06	FALSE	1;2
DMC1	DNA meiotic recombinase 1	Hs.339396	-0,67	5,65E-05	8,34E-03	FALSE	-0,28	1,85E-05	2,22E-04	FALSE	1;2
APOBEC3C	apolipoprotein B mRNA editing enzyme, catalytic polypeptide-like 3C	Hs.441124	-0,67	5,92E-05	8,43E-03	FALSE	-0,59	0,00E+00	0,00E+00	FALSE	0;0
HNF4A	hepatocyte nuclear factor 4, alpha	Hs.116462	-0,67	6,01E-05	8,43E-03	FALSE	-0,44	8,89E-12	3,34E-10	FALSE	0;1
MAP1LC3A	microtubule-associated protein 1 light chain 3 alpha		-0,66	6,20E-05	8,59E-03	FALSE	-0,30	4,54E-06	6,36E-05	FALSE	0;2
MIA	melanoma inhibitory activity	Hs.646364	-0,66	6,30E-05	8,62E-03	FALSE	-0,51	4,44E-16	2,88E-14	FALSE	0;0
MLPH	melanophilin	Hs.102406	-0,66	7,45E-05	9,84E-03	FALSE	-0,45	2,90E-12	1,16E-10	FALSE	0;1
TPM2	tropomyosin 2 (beta)	Hs.300772	-0,66	8,15E-05	1,04E-02	FALSE	-0,46	5,60E-13	2,51E-11	FALSE	0;0
KCNE3	potassium channel, voltage gated subfamily E regulatory beta subunit 3	Hs.523899	-0,66	8,15E-05	1,04E-02	FALSE	-0,30	5,54E-06	7,64E-05	FALSE	1;1
RPL39L	ribosomal protein L39-like	Hs.647900	-0,66	8,28E-05	1,04E-02	FALSE	-0,71	0,00E+00	0,00E+00	FALSE	1;1
GSTT1	glutathione S-transferase theta 1	Hs.268573	-0,65	8,92E-05	1,05E-02	FALSE	-0,25	2,03E-04	1,78E-03	FALSE	0;0
TNFRSF10C	tumor necrosis factor receptor superfamily, member 10c, decoy without an intracellular domain	Hs.655801	-0,65	8,92E-05	1,05E-02	FALSE	-0,39	2,47E-09	6,47E-08	FALSE	0;1
HOXB6	homeobox B6	Hs.98428	-0,65	8,92E-05	1,05E-02	FALSE	-0,61	0,00E+00	0,00E+00	FALSE	1;2
STK33	serine/threonine kinase 33	Hs.501833	-0,65	9,18E-05	1,06E-02	FALSE	-0,43	2,24E-11	7,79E-10	FALSE	1;2
CDH17	cadherin 17, LI cadherin (liver-intestine)	Hs.591853	-0,65	1,09E-04	1,21E-02	FALSE	-0,12	7,51E-02	1,92E-01	FALSE	0;0
STAP2	signal transducing adaptor family member 2	Hs.194385	-0,65	1,09E-04	1,21E-02	FALSE	-0,18	7,90E-03	3,48E-02	FALSE	1;2
HOXB3	homeobox B3	Hs.654560	-0,65	1,13E-04	1,21E-02	FALSE	-0,54	0,00E+00	0,00E+00	FALSE	2;5
AMIGO2	adhesion molecule with Ig-like domain 2	Hs.121520	-0,64	1,21E-04	1,26E-02	FALSE	-0,15	2,13E-02	7,56E-02	FALSE	1;1
MYCN	v-myc avian myelocytomatosis viral oncogene neuroblastoma derived homolog	Hs.25960	-0,64	1,28E-04	1,31E-02	TRUE	-0,24	3,06E-04	2,48E-03	FALSE	1;1
PPP1R1B	protein phosphatase 1, regulatory (inhibitor) subunit 1B (dopamine and cAMP regulated phosphoprotein, DARPP-32)		-0,64	1,30E-04	1,32E-02	FALSE	-0,12	6,31E-02	1,69E-01	FALSE	1;1
LXN	latexin	Hs.478067	-0,64	1,34E-04	1,33E-02	FALSE	-0,18	7,54E-03	3,36E-02	FALSE	0;0
GSTM3	glutathione S-transferase mu 3 (brain)	Hs.2006	-0,64	1,36E-04	1,34E-02	FALSE	-0,38	6,03E-09	1,48E-07	FALSE	0;1
MT2A	metallothionein 2A	Hs.534330	-0,64	1,42E-04	1,38E-02	FALSE	-0,21	1,89E-03	1,11E-02	FALSE	1;1
SLC35D2	solute carrier family 35 (UDP-GlcNAc/UDP-glucose transporter), member D2	Hs.494556	-0,64	1,48E-04	1,41E-02	FALSE	-0,20	2,31E-03	1,32E-02	FALSE	1;3
KLK1	kallikrein 1	Hs.123107	-0,64	1,58E-04	1,47E-02	FALSE	-0,31	3,27E-06	4,77E-05	FALSE	0;1
CMTM3	CKLF-like MARVEL transmembrane domain containing 3	Hs.298198	-0,63	1,72E-04	1,56E-02	FALSE	-0,47	1,29E-13	6,28E-12	FALSE	0;0
CKB	creatine kinase, brain	Hs.173724	-0,63	1,72E-04	1,56E-02	FALSE	-0,25	1,32E-04	1,25E-03	FALSE	1;1
LIME1	Lck interacting transmembrane adaptor 1	Hs.233220	-0,63	1,77E-04	1,58E-02	FALSE	-0,20	2,47E-03	1,40E-02	FALSE	1;1
THBD	thrombomodulin	Hs.2030	-0,63	1,77E-04	1,58E-02	FALSE	-0,33	4,81E-07	8,18E-06	FALSE	1;1
HCP5	HLA complex P5 (non-protein coding)	Hs.691948	-0,63	1,84E-04	1,62E-02	FALSE	-0,19	3,55E-03	1,86E-02	FALSE	0;0
BMP7	bone morphogenetic protein 7	Hs.473163	-0,63	1,89E-04	1,63E-02	FALSE	-0,57	0,00E+00	0,00E+00	FALSE	1;1
CBLC	Cbl proto-oncogene C, E3 ubiquitin protein ligase	Hs.466907	-0,63	1,92E-04	1,64E-02	FALSE	-0,23	7,00E-04	4,98E-03	FALSE	0;1
CADPS	Ca++-dependent secretion activator	Hs.654933	-0,63	2,00E-04	1,68E-02	FALSE	-0,59	0,00E+00	0,00E+00	FALSE	1;2
PDZK1	PDZ domain containing 1	Hs.444751	-0,63	2,11E-04	1,72E-02	TRUE	-0,44	4,30E-12	1,67E-10	FALSE	0;1
SNTB1	syntrophin, beta 1 (dystrophin-associated protein A1, 59kDa, basic component 1)	Hs.46701	-0,62	2,29E-04	1,84E-02	FALSE	-0,37	1,50E-08	3,41E-07	FALSE	1;2
ZNF350	zinc finger protein 350	Hs.407694	-0,62	2,32E-04	1,85E-02	FALSE	-0,58	0,00E+00	0,00E+00	FALSE	0;0
GALC	galactosylceramidase	Hs.513439	-0,62	2,38E-04	1,89E-02	FALSE	-0,56	0,00E+00	0,00E+00	FALSE	1;1
KRT20	keratin 20, type I	Hs.84905	-0,62	2,44E-04	1,91E-02	FALSE	-0,26	6,66E-05	6,89E-04	FALSE	0;0
ZNF223	zinc finger protein 223	Hs.720813	-0,62	2,48E-04	1,91E-02	FALSE	-0,14	3,46E-02	1,09E-01	FALSE	0;1
DAPP1	dual adaptor of phosphotyrosine and 3-phosphoinositides	Hs.436271	-0,62	2,51E-04	1,93E-02	FALSE	-0,77	0,00E+00	0,00E+00	FALSE	0;0

Gene ID	Name	UGCluster	Spearman r (cell lines)	Spearman p (cell lines)	FDR adjusted (cell lines)	L-shape (cell lines)	Spearman r (primay tumors)	Spearman p (primay tumors)	FDR adjusted (primay tumors)	L-shape (primay tumors)	CpG islands (promoter;intragenic)[1]
BCL2L14	BCL2-like 14 (apoptosis facilitator)	Hs.210343	-0,62	2,58E-04	1,96E-02	FALSE	-0,20	2,14E-03	1,24E-02	FALSE	0;0
PTK6	protein tyrosine kinase 6	Hs.51133	-0,62	2,75E-04	2,07E-02	FALSE	-0,20	3,07E-03	1,65E-02	FALSE	0;2
DNAJA4	DnaJ (Hsp40) homolog, subfamily A, member 4	Hs.513053	-0,62	2,75E-04	2,07E-02	FALSE	-0,21	1,45E-03	9,00E-03	FALSE	1;1
DAPK2	death-associated protein kinase 2	Hs.237886	-0,62	2,94E-04	2,17E-02	FALSE	-0,23	6,53E-04	4,71E-03	FALSE	1;1
ARL14	ADP-ribosylation factor-like 14	Hs.287702	-0,62	2,98E-04	2,17E-02	FALSE	-0,21	1,28E-03	8,06E-03	FALSE	0;0
PSTPIP2	proline-serine-threonine phosphatase interacting protein 2	Hs.567384	-0,62	2,98E-04	2,17E-02	FALSE	-0,17	1,17E-02	4,72E-02	FALSE	1;2
PON3	paraoxonase 3	Hs.440967	-0,61	3,17E-04	2,24E-02	FALSE	-0,30	3,68E-06	5,30E-05	FALSE	1;1
PDE3B	phosphodiesterase 3B, cGMP-inhibited	Hs.445711	-0,61	3,38E-04	2,34E-02	FALSE	-0,24	3,85E-04	3,01E-03	FALSE	1;3
HOXB5	homeobox B5	Hs.654456	-0,61	3,38E-04	2,34E-02	FALSE	-0,45	1,19E-12	5,11E-11	FALSE	3;2
ACSL5	acyl-CoA synthetase long-chain family member 5	Hs.11638	-0,61	3,43E-04	2,36E-02	FALSE	-0,52	0,00E+00	0,00E+00	FALSE	0;1
LY6K	lymphocyte antigen 6 complex, locus K	Hs.69517	-0,61	3,56E-04	2,42E-02	FALSE	-0,12	6,31E-02	1,69E-01	FALSE	0;1
CHCHD5	coiled-coil-helix-coiled-coil-helix domain containing 5	Hs.375707	-0,61	3,61E-04	2,42E-02	FALSE	-0,15	2,57E-02	8,70E-02	FALSE	1;1
MMP15	matrix metallopeptidase 15 (membrane-inserted)	Hs.80343	-0,61	3,61E-04	2,42E-02	FALSE	-0,15	2,36E-02	8,17E-02	FALSE	1;3
TIAM1	T-cell lymphoma invasion and metastasis 1	Hs.517228	-0,61	3,61E-04	2,42E-02	FALSE	-0,38	4,36E-09	1,09E-07	FALSE	1;5
KLF7	Kruppel-like factor 7 (ubiquitous)	Hs.59908	-0,61	3,74E-04	2,46E-02	FALSE	-0,25	1,74E-04	1,57E-03	FALSE	0;0
ZNF345	zinc finger protein 345	Hs.362324	-0,61	3,79E-04	2,46E-02	FALSE	-0,53	0,00E+00	0,00E+00	FALSE	1;2
ABCC2	ATP-binding cassette, sub-family C (CFTR/MRP), member 2	Hs.368243	-0,61	3,84E-04	2,46E-02	FALSE	-0,71	0,00E+00	0,00E+00	FALSE	0;1
EPDR1	ependymin related 1	Hs.563491	-0,61	3,94E-04	2,48E-02	FALSE	-0,47	7,42E-14	3,80E-12	FALSE	1;1
YBX2	Y box binding protein 2	Hs.567494	-0,61	3,94E-04	2,48E-02	FALSE	-0,19	5,37E-03	2,57E-02	FALSE	2;2
S100A4	S100 calcium binding protein A4	Hs.654444	-0,60	4,04E-04	2,52E-02	FALSE	-0,60	0,00E+00	0,00E+00	FALSE	0;0
SLC7A7	solute carrier family 7 (amino acid transporter light chain, y+L system), member 7	Hs.513147	-0,60	4,09E-04	2,54E-02	FALSE	-0,29	7,45E-06	1,00E-04	FALSE	4;0
EREG	epiregulin	Hs.115263	-0,60	4,24E-04	2,58E-02	FALSE	-0,34	2,38E-07	4,33E-06	FALSE	1;0
ISYNA1	inositol-3-phosphate synthase 1	Hs.405873	-0,60	4,24E-04	2,58E-02	FALSE	-0,15	2,16E-02	7,64E-02	FALSE	1;1
NUDT12	nudix (nucleoside diphosphate linked moiety X)-type motif 12	Hs.434289	-0,60	4,40E-04	2,65E-02	FALSE	-0,49	9,77E-15	5,43E-13	FALSE	0;0
SLC39A5	solute carrier family 39 (zinc transporter), member 5	Hs.591018	-0,60	4,46E-04	2,65E-02	FALSE	-0,48	2,26E-14	1,20E-12	FALSE	0;0
ADD3	adducin 3 (gamma)	Hs.501012	-0,60	4,46E-04	2,65E-02	FALSE	-0,35	6,77E-08	1,36E-06	FALSE	0;1
TRERF1	transcriptional regulating factor 1	Hs.485392	-0,60	4,51E-04	2,66E-02	FALSE	-0,49	8,22E-15	4,61E-13	FALSE	1;3
HOXB8	homeobox B8	Hs.514292	-0,60	4,51E-04	2,66E-02	FALSE	-0,47	1,15E-13	5,67E-12	FALSE	2;1
EHF	ets homologous factor	Hs.653859	-0,60	4,63E-04	2,68E-02	FALSE	-0,14	3,11E-02	1,01E-01	FALSE	0;0
ST6GALNAC:	ST6 (alpha-N-acetyl-neuraminyl-2,3-beta-galactosyl-1,3)-N-acetylgalactosaminide alpha-2,6-sialyltransferase 1	Hs.105352	-0,60	4,63E-04	2,68E-02	FALSE	-0,13	6,21E-02	1,67E-01	FALSE	0;2
VAMP5	vesicle-associated membrane protein 5	Hs.534373	-0,60	4,80E-04	2,77E-02	FALSE	-0,20	2,13E-03	1,24E-02	FALSE	1;1
FOXA3	forkhead box A3	Hs.36137	-0,60	4,86E-04	2,77E-02	FALSE	-0,14	4,30E-02	1,29E-01	FALSE	1;1
FUT2	fucosyltransferase 2 (secretor status included)	Hs.579928	-0,60	4,98E-04	2,80E-02	FALSE	-0,19	4,83E-03	2,37E-02	FALSE	0;2
ATP2A3	ATPase, Ca++ transporting, ubiquitous	Hs.513870	-0,60	4,98E-04	2,80E-02	TRUE	-0,20	2,60E-03	1,45E-02	FALSE	1;5
SERPINB1	serpin peptidase inhibitor, clade B (ovalbumin), member 1	Hs.381167	-0,59	5,29E-04	2,90E-02	FALSE	-0,18	7,71E-03	3,43E-02	FALSE	0;1
REG4	regenerating islet-derived family, member 4	Hs.660883	-0,59	5,55E-04	3,00E-02	FALSE	-0,19	4,64E-03	2,29E-02	FALSE	0;0
PLEKHG6	pleckstrin homology domain containing, family G (with RhoGef domain) member 6	Hs.631660	-0,59	5,55E-04	3,00E-02	FALSE	-0,16	1,67E-02	6,25E-02	FALSE	1;2
ZNF585A	zinc finger protein 585A	Hs.659236	-0,59	5,69E-04	3,03E-02	FALSE	-0,22	8,85E-04	6,02E-03	FALSE	0;1
C1QTNF6	C1q and tumor necrosis factor related protein 6	Hs.22011	-0,59	5,76E-04	3,05E-02	FALSE	-0,16	1,71E-02	6,38E-02	FALSE	0;1
MAP2K6	mitogen-activated protein kinase kinase 6	Hs.463978	-0,59	5,83E-04	3,05E-02	FALSE	-0,21	1,70E-03	1,02E-02	FALSE	0;2
NES	nestin	Hs.527971	-0,59	5,83E-04	3,05E-02	FALSE	-0,21	1,28E-03	8,09E-03	FALSE	1;1
NME4	NME/NM23 nucleoside diphosphate kinase 4	Hs.9235	-0,59	5,90E-04	3,07E-02	FALSE	-0,35	9,60E-08	1,88E-06	FALSE	0;1
ITGB6	integrin, beta 6	Hs.470399	-0,59	6,04E-04	3,13E-02	FALSE	-0,17	1,25E-02	4,98E-02	FALSE	0;0
ZNF573	zinc finger protein 573	Hs.531262	-0,59	6,18E-04	3,18E-02	FALSE	-0,14	4,17E-02	1,26E-01	FALSE	1;0
RNASE1	ribonuclease, RNase A family, 1 (pancreatic)	Hs.78224	-0,59	6,41E-04	3,25E-02	FALSE	-0,13	6,02E-02	1,64E-01	FALSE	0;0
SELENBP1	selenium binding protein 1	Hs.632460	-0,59	6,41E-04	3,25E-02	FALSE	-0,37	7,75E-09	1,87E-07	FALSE	0;0
ACY1	aminoacylase 1	Hs.334707	-0,59	6,48E-04	3,26E-02	FALSE	-0,58	0,00E+00	0,00E+00	FALSE	1;1
ANXA13	annexin A13	Hs.181107	-0,58	6,88E-04	3,37E-02	FALSE	-0,35	6,92E-08	1,39E-06	FALSE	0;1
MYD88	myeloid differentiation primary response 88	Hs.82116	-0,58	7,64E-04	3,71E-02	FALSE	-0,20	3,14E-03	1,68E-02	FALSE	2;1
ZNF426	zinc finger protein 426	Hs.594011	-0,58	7,90E-04	3,80E-02	FALSE	-0,40	4,09E-10	1,21E-08	FALSE	1;2
CARD10	caspase recruitment domain family, member 10	Hs.57973	-0,58	8,57E-04	4,00E-02	FALSE	-0,19	4,64E-03	2,29E-02	FALSE	1;2
NMU	neuromedin U	Hs.418367	-0,58	8,66E-04	4,03E-02	FALSE	-0,28	2,65E-05	3,06E-04	FALSE	1;3
RPH3AL	rabphilin 3A-like (without C2 domains)	Hs.651925	-0,58	8,86E-04	4,09E-02	FALSE	-0,39	1,55E-09	4,17E-08	FALSE	1;14
AKR7A3	aldo-keto reductase family 7, member A3 (aflatoxin aldehyde reductase)	Hs.6980	-0,57	9,17E-04	4,20E-02	FALSE	-0,17	9,67E-03	4,08E-02	FALSE	1;1
CST7	cystatin F (leukocystatin)	Hs.143212	-0,57	9,27E-04	4,21E-02	FALSE	-0,35	8,89E-08	1,75E-06	FALSE	0;0
TRMT12	tRNA methyltransferase 12 homolog (S. cerevisiae)	Hs.9925	-0,57	9,27E-04	4,21E-02	FALSE	-0,52	0,00E+00	0,00E+00	FALSE	0;0
SULT1A2	sulfotransferase family, cytosolic, 1A, phenol-preferring, member 2	Hs.546304	-0,57	9,38E-04	4,24E-02	FALSE	-0,22	9,83E-04	6,57E-03	FALSE	1;0
TFAP2C	transcription factor AP-2 gamma (activating enhancer binding protein 2 gamma)	Hs.473152	-0,57	9,70E-04	4,37E-02	FALSE	-0,40	4,54E-10	1,32E-08	FALSE	1;1
EPM2AIP1	EPM2A (laforin) interacting protein 1	Hs.28020	-0,57	1,03E-03	4,59E-02	FALSE	-0,33	4,20E-07	7,24E-06	FALSE	1;1
SULF2	sulfatase 2	Hs.162016	-0,57	1,04E-03	4,60E-02	FALSE	-0,15	2,75E-02	9,15E-02	FALSE	1;2
WFDC2	WAP four-disulfide core domain 2	Hs.2719	-0,57	1,06E-03	4,67E-02	TRUE	-0,27	4,11E-05	4,53E-04	FALSE	1;1
IGF1R	insulin-like growth factor 1 receptor	Hs.643120	-0,57	1,06E-03	4,67E-02	FALSE	-0,12	7,87E-02	1,98E-01	FALSE	1;5
TMED6	transmembrane emp24 protein transport domain containing 6	Hs.729118	-0,57	1,10E-03	4,68E-02	TRUE	-0,15	2,86E-02	9,44E-02	FALSE	0;0
DFNA5	deafness, autosomal dominant 5	Hs.520708	-0,57	1,10E-03	4,68E-02	FALSE	-0,12	6,52E-02	1,73E-01	FALSE	0;1
PLA2G2A	phospholipase A2, group IIA (platelets, synovial fluid)	Hs.466804	-0,57	1,12E-03	4,75E-02	FALSE	-0,38	7,26E-09	1,75E-07	FALSE	0;0
CHRNB1	cholinergic receptor, nicotinic, beta 1 (muscle)	Hs.330386	-0,57	1,13E-03	4,79E-02	FALSE	-0,20	2,55E-03	1,43E-02	FALSE	2;3
ZNF597	zinc finger protein 597	Hs.88630	-0,56	1,16E-03	4,82E-02	FALSE	-0,25	1,64E-04	1,49E-03	FALSE	1;1
CCND2	cyclin D2	Hs.376071	-0,56	1,20E-03	4,93E-02	FALSE	-0,30	4,01E-06	5,71E-05	FALSE	1;1
ARL11	ADP-ribosylation factor-like 11	Hs.558599	-0,56	1,21E-03	4,96E-02	FALSE	-0,62	0,00E+00	0,00E+00	FALSE	0;0
ALDH1A3	aldehyde dehydrogenase 1 family, member A3	Hs.459538	-0,56	1,24E-03	5,00E-02	FALSE	-0,29	1,38E-05	1,73E-04	FALSE	1;2
HBQ1	hemoglobin, theta 1	Hs.247921	-0,56	1,24E-03	5,00E-02	FALSE	-0,12	7,86E-02	1,98E-01	FALSE	2;1
GALNT14	polypeptide N-acetylgalactosaminyltransferase 14	Hs.468058	-0,56	1,25E-03	5,02E-02	FALSE	-0,24	3,09E-04	2,50E-03	FALSE	1;2
OAS2	2'-5'-oligoadenylate synthetase 2, 69/71kDa	Hs.414332	-0,56	1,31E-03	5,13E-02	FALSE	-0,49	4,88E-15	2,82E-13	FALSE	0;0
PPP1R9A	protein phosphatase 1, regulatory subunit 9A	Hs.21816	-0,56	1,31E-03	5,13E-02	FALSE	-0,54	0,00E+00	0,00E+00	FALSE	1;5
RALGDS	ral guanine nucleotide dissociation stimulator	Hs.106185	-0,56	1,33E-03	5,19E-02	FALSE	-0,12	7,36E-02	1,88E-01	FALSE	1;1
IL1RL2	interleukin 1 receptor-like 2	Hs.659863	-0,56	1,35E-03	5,21E-02	FALSE	-0,22	1,23E-03	7,82E-03	FALSE	0;1
ADAM19	ADAM metallopeptidase domain 19	Hs.483944	-0,56	1,39E-03	5,33E-02	FALSE	-0,23	7,10E-04	5,03E-03	FALSE	0;0
CYP27A1	cytochrome P450, family 27, subfamily A, polypeptide 1	Hs.516700	-0,56	1,39E-03	5,33E-02	FALSE	-0,20	2,84E-03	1,55E-02	FALSE	2;1
PLAGL1	pleiomorphic adenoma gene-like 1	Hs.743225	-0,56	1,41E-03	5,35E-02	FALSE	-0,46	4,80E-13	2,16E-11	FALSE	0;1
SH3TC2	SH3 domain and tetratricopeptide repeats 2	Hs.483784	-0,56	1,44E-03	5,43E-02	FALSE	-0,55	0,00E+00	0,00E+00	FALSE	0;0
RAB3D	RAB3D, member RAS oncogene family	Hs.744916	-0,55	1,47E-03	5,47E-02	FALSE	-0,19	3,77E-03	1,95E-02	FALSE	1;1
KRTCAP3	keratinocyte associated protein 3	Hs.59509	-0,55	1,57E-03	5,67E-02	FALSE	-0,13	4,91E-02	1,41E-01	FALSE	1;1
TRAK1	trafficking protein, kinesin binding 1	Hs.535711	-0,55	1,60E-03	5,73E-02	FALSE	-0,16	1,55E-02	5,91E-02	FALSE	0;1
DQX1	DEAQ box RNA-dependent ATPase 1	Hs.191705	-0,55	1,60E-03	5,73E-02	FALSE	-0,70	0,00E+00	0,00E+00	FALSE	1;0
ABO	ABO blood group (transferase A, alpha 1-3-N-acetylgalactosaminyltransferase; transferase B, alpha 1-3-galactosyltransferase)	Hs.654423	-0,55	1,62E-03	5,76E-02	FALSE	-0,15	2,10E-02	7,47E-02	FALSE	1;2
PF4	platelet factor 4	Hs.81564	-0,55	1,65E-03	5,81E-02	FALSE	-0,49	4,44E-15	2,60E-13	FALSE	0;1
FAM84A	family with sequence similarity 84, member A	Hs.260855	-0,55	1,65E-03	5,81E-02	TRUE	-0,23	4,16E-04	3,21E-03	FALSE	1;1
SPINK1	serine peptidase inhibitor, Kazal type 1	Hs.407856	-0,55	1,67E-03	5,83E-02	FALSE	-0,27	3,96E-05	4,38E-04	FALSE	0;0
MST1R	macrophage stimulating 1 receptor	Hs.517973	-0,55	1,67E-03	5,83E-02	FALSE	-0,23	4,11E-04	3,17E-03	FALSE	1;2

Gene ID	Name	UGCluster	Spearman r (cell lines)	Spearman p (cell lines)	FDR adjusted (cell lines)	L-shape (cell lines)	Spearman r (primay tumors)	Spearman p (primay tumors)	FDR adjusted (primay tumors)	L-shape (primay tumors)	CpG islands (promoter;intragenic)[1]
TMEM25	transmembrane protein 25	Hs.564188	-0,55	1,72E-03	5,93E-02	FALSE	-0,57	0,00E+00	0,00E+00	FALSE	1;1
ZNF215	zinc finger protein 215	Hs.523457	-0,55	1,74E-03	5,95E-02	FALSE	-0,35	7,22E-08	1,44E-06	FALSE	1;1
PCK1	phosphoenolpyruvate carboxykinase 1 (soluble)	Hs.1872	-0,55	1,76E-03	5,96E-02	FALSE	-0,21	1,27E-03	8,03E-03	FALSE	0;0
SLC22A3	solute carrier family 22 (organic cation transporter), member 3	Hs.567337	-0,55	1,76E-03	5,96E-02	FALSE	-0,28	2,20E-05	2,59E-04	FALSE	1;2
ZNF530	zinc finger protein 530	Hs.97111	-0,55	1,81E-03	6,11E-02	FALSE	-0,56	0,00E+00	0,00E+00	FALSE	0;0
ZNF655	zinc finger protein 655	Hs.599798	-0,54	1,87E-03	6,21E-02	FALSE	-0,71	0,00E+00	0,00E+00	FALSE	0;0
MLH1	mutL homolog 1	Hs.195364	-0,54	1,91E-03	6,27E-02	FALSE	-0,43	1,51E-11	5,45E-10	FALSE	1;1
ITGA9	integrin, alpha 9	Hs.113157	-0,54	1,91E-03	6,27E-02	FALSE	-0,39	2,05E-09	5,42E-08	FALSE	1;2
SYT7	synaptotagmin VII	Hs.131188	-0,54	1,93E-03	6,32E-02	FALSE	-0,43	1,32E-11	4,83E-10	FALSE	1;6
RORC	RAR-related orphan receptor C	Hs.256022	-0,54	1,97E-03	6,37E-02	FALSE	-0,54	0,00E+00	0,00E+00	FALSE	0;0
PCDHB2	protocadherin beta 2	Hs.533023	-0,54	1,99E-03	6,40E-02	FALSE	-0,54	0,00E+00	0,00E+00	FALSE	0;1
RASGRF2	Ras protein-specific guanine nucleotide-releasing factor 2	Hs.162129	-0,54	1,99E-03	6,40E-02	TRUE	-0,14	4,11E-02	1,24E-01	FALSE	1;2
HKDC1	hexokinase domain containing 1	Hs.522988	-0,54	2,03E-03	6,46E-02	FALSE	-0,32	8,77E-07	1,44E-05	FALSE	0;2
PRTFDC1	phosphoribosyl transferase domain containing 1	Hs.405619	-0,54	2,03E-03	6,46E-02	FALSE	-0,19	4,50E-03	2,24E-02	FALSE	1;2
ZNF566	zinc finger protein 566	Hs.533939	-0,54	2,03E-03	6,46E-02	FALSE	-0,22	1,04E-03	6,87E-03	FALSE	1;2
ELF5	E74-like factor 5 (ets domain transcription factor)	Hs.11713	-0,54	2,09E-03	6,57E-02	FALSE	-0,67	0,00E+00	0,00E+00	FALSE	0;0
EMP2	epithelial membrane protein 2	Hs.531561	-0,54	2,09E-03	6,57E-02	FALSE	-0,16	1,73E-02	6,44E-02	FALSE	1;1
DNM1L	dynamin 1-like	Hs.556296	-0,54	2,14E-03	6,61E-02	FALSE	-0,23	5,40E-04	4,02E-03	FALSE	1;2
ABCC13	ATP-binding cassette, sub-family C (CFTR/MRP), member 13, pseudogene	Hs.366575	-0,54	2,22E-03	6,76E-02	FALSE	-0,30	4,76E-06	6,65E-05	FALSE	0;0
SCNN1A	sodium channel, non voltage gated 1 alpha subunit	Hs.591047	-0,54	2,27E-03	6,82E-02	FALSE	-0,60	0,00E+00	0,00E+00	FALSE	0;3
SLC5A1	solute carrier family 5 (sodium/glucose cotransporter), member 1	Hs.1964	-0,53	2,34E-03	6,99E-02	FALSE	-0,14	3,23E-02	1,04E-01	FALSE	0;1
AKR1B1	aldo-keto reductase family 1, member B1 (aldose reductase)	Hs.521212	-0,53	2,43E-03	7,22E-02	FALSE	-0,44	4,47E-12	1,73E-10	FALSE	1;1
TMC7	transmembrane channel-like 7	Hs.187377	-0,53	2,51E-03	7,36E-02	FALSE	-0,27	6,03E-05	6,34E-04	FALSE	1;1
IL1RN	interleukin 1 receptor antagonist	Hs.81134	-0,53	2,53E-03	7,38E-02	FALSE	-0,31	3,01E-06	4,42E-05	FALSE	0;0
ZNF551	zinc finger protein 551	Hs.109540	-0,53	2,56E-03	7,41E-02	FALSE	-0,38	3,57E-09	9,13E-08	FALSE	1;1
USH1C	Usher syndrome 1C (autosomal recessive, severe)		-0,53	2,58E-03	7,45E-02	FALSE	-0,19	5,53E-03	2,63E-02	FALSE	1;1
NAV2	neuron navigator 2	Hs.64341	-0,53	2,58E-03	7,45E-02	FALSE	-0,23	4,98E-04	3,75E-03	FALSE	1;4
SUOX	sulfite oxidase	Hs.558403	-0,53	2,66E-03	7,60E-02	FALSE	-0,30	7,39E-06	9,92E-05	FALSE	0;2
FBXO2	F-box protein 2	Hs.132753	-0,53	2,66E-03	7,60E-02	FALSE	-0,70	0,00E+00	0,00E+00	FALSE	1;3
PGBD4	piggyBac transposable element derived 4	Hs.156317	-0,53	2,71E-03	7,69E-02	FALSE	-0,32	1,36E-06	2,14E-05	FALSE	2;0
PPARA	peroxisome proliferator-activated receptor alpha	Hs.103110	-0,53	2,82E-03	7,92E-02	FALSE	-0,27	4,04E-05	4,47E-04	FALSE	1;2
TCF4	transcription factor 4	Hs.605153	-0,53	2,85E-03	7,94E-02	FALSE	-0,14	3,30E-02	1,06E-01	FALSE	1;4
ZNF532	zinc finger protein 532	Hs.529023	-0,52	2,91E-03	7,99E-02	FALSE	-0,20	2,17E-03	1,26E-02	FALSE	0;2
MYO1A	myosin IA	Hs.5394	-0,52	2,93E-03	8,05E-02	FALSE	-0,34	1,93E-07	3,56E-06	FALSE	0;0
MT1E	metallothionein 1E	Hs.744893	-0,52	3,02E-03	8,19E-02	FALSE	-0,44	8,29E-12	3,12E-10	FALSE	1;1
MNS1	meiosis-specific nuclear structural 1	Hs.444483	-0,52	3,05E-03	8,21E-02	FALSE	-0,34	1,65E-07	3,10E-06	FALSE	1;1
FABP1	fatty acid binding protein 1, liver	Hs.380135	-0,52	3,11E-03	8,31E-02	FALSE	-0,38	4,12E-09	1,04E-07	FALSE	0;0
C10orf82	chromosome 10 open reading frame 82	Hs.121347	-0,52	3,11E-03	8,31E-02	FALSE	-0,75	0,00E+00	0,00E+00	FALSE	1;1
ZNF544	zinc finger protein 544	Hs.438994	-0,52	3,14E-03	8,35E-02	FALSE	-0,65	0,00E+00	0,00E+00	FALSE	0;0
HSPA2	heat shock 70kDa protein 2	Hs.432648	-0,52	3,14E-03	8,35E-02	FALSE	-0,35	6,35E-08	1,28E-06	FALSE	1;1
USP5	ubiquitin specific peptidase 5 (isopeptidase T)	Hs.631661	-0,52	3,26E-03	8,60E-02	FALSE	-0,20	2,51E-03	1,42E-02	FALSE	1;2
TMEM92	transmembrane protein 92	Hs.224630	-0,52	3,33E-03	8,68E-02	FALSE	-0,30	3,66E-06	5,28E-05	FALSE	0;1
CXADR	coxsackie virus and adenovirus receptor	Hs.627078	-0,52	3,33E-03	8,68E-02	FALSE	-0,28	1,62E-05	1,97E-04	FALSE	1;1
C15orf48	chromosome 15 open reading frame 48	Hs.112242	-0,52	3,39E-03	8,83E-02	FALSE	-0,15	2,73E-02	9,08E-02	FALSE	1;1
GLDC	glycine dehydrogenase (decarboxylating)	Hs.584238	-0,52	3,45E-03	8,94E-02	FALSE	-0,47	1,13E-13	5,59E-12	FALSE	2;3
C10orf99	chromosome 10 open reading frame 99	Hs.298713	-0,51	3,62E-03	9,29E-02	FALSE	-0,28	2,39E-05	2,80E-04	FALSE	0;0
PTPN22	protein tyrosine phosphatase, non-receptor type 22 (lymphoid)	Hs.535276	-0,51	3,62E-03	9,29E-02	FALSE	-0,37	8,51E-09	2,02E-07	FALSE	0;2
DOLPP1	dolichyldiphosphatase 1	Hs.21701	-0,51	3,69E-03	9,38E-02	FALSE	-0,13	4,54E-02	1,33E-01	FALSE	1;1
PER3	period circadian clock 3	Hs.162200	-0,51	3,69E-03	9,38E-02	FALSE	-0,16	1,99E-02	7,17E-02	FALSE	1;3
PLA2R1	phospholipase A2 receptor 1, 180kDa	Hs.410477	-0,51	3,69E-03	9,38E-02	FALSE	-0,29	1,32E-05	1,66E-04	FALSE	1;3
NUDT15	nudix (nucleoside diphosphate linked moiety X)-type motif 15	Hs.144407	-0,51	3,76E-03	9,45E-02	FALSE	-0,20	3,27E-03	1,74E-02	FALSE	1;2
CTTNBP2	cortactin binding protein 2	Hs.592285	-0,51	3,80E-03	9,48E-02	FALSE	-0,42	5,33E-11	1,79E-09	FALSE	1;2
ABCB6	ATP-binding cassette, sub-family B (MDR/TAP), member 6 (Langereis blood group)	Hs.107911	-0,51	3,87E-03	9,57E-02	FALSE	-0,15	2,23E-02	7,82E-02	FALSE	2;1
CENPB	centromere protein B, 80kDa	Hs.516855	-0,51	3,90E-03	9,62E-02	FALSE	-0,19	4,48E-03	2,23E-02	FALSE	1;1
ICAM4	intercellular adhesion molecule 4 (Landsteiner-Wiener blood group)	Hs.706750	-0,51	3,90E-03	9,62E-02	FALSE	-0,25	1,20E-04	1,15E-03	FALSE	1;1
HIST1H3C	histone cluster 1, H3c	Hs.248176	-0,51	3,94E-03	9,65E-02	FALSE	-0,12	6,64E-02	1,76E-01	FALSE	3;1
ELMO3	engulfment and cell motility 3	Hs.377416	-0,51	3,98E-03	9,65E-02	FALSE	-0,14	4,00E-02	1,22E-01	FALSE	1;1
GGT6	gamma-glutamyltransferase 6	Hs.130749	-0,51	4,05E-03	9,77E-02	FALSE	-0,36	3,71E-08	7,86E-07	FALSE	0;0
TLR4	toll-like receptor 4	Hs.174312	-0,51	4,09E-03	9,77E-02	FALSE	-0,16	1,86E-02	6,80E-02	FALSE	0;0
AQP5	aquaporin 5	Hs.298023	-0,51	4,09E-03	9,77E-02	FALSE	-0,20	2,80E-03	1,54E-02	FALSE	1;1
ASPHD1	aspartate beta-hydroxylase domain containing 1	Hs.655761	-0,51	4,09E-03	9,77E-02	FALSE	-0,44	3,86E-12	1,51E-10	FALSE	1;1
SLC35E4	solute carrier family 35, member E4	Hs.660384	-0,51	4,09E-03	9,77E-02	FALSE	-0,15	2,24E-02	7,84E-02	FALSE	2;1
HNMT	histamine N-methyltransferase	Hs.42151	-0,51	4,13E-03	9,77E-02	FALSE	-0,31	2,56E-06	3,81E-05	FALSE	0;1
HOXB2	homeobox B2	Hs.514289	-0,51	4,13E-03	9,77E-02	FALSE	-0,49	9,10E-15	5,09E-13	FALSE	0;1
H2AFY2	H2A histone family, member Y2	Hs.499953	-0,51	4,13E-03	9,77E-02	FALSE	-0,47	9,28E-14	4,70E-12	FALSE	1;2
ZNF613	zinc finger protein 613	Hs.183390	-0,51	4,17E-03	9,84E-02	FALSE	-0,18	5,95E-03	2,77E-02	FALSE	0;0
SLC1A4	solute carrier family 1 (glutamate/neutral amino acid transporter), member 4	Hs.654352	-0,51	4,21E-03	9,87E-02	FALSE	-0,20	2,30E-03	1,32E-02	FALSE	0;0
TMEM45B	transmembrane protein 45B	Hs.504301	-0,51	4,21E-03	9,87E-02	FALSE	-0,28	1,80E-05	2,17E-04	FALSE	1;2
IL23A	interleukin 23, alpha subunit p19	Hs.98309	-0,51	4,24E-03	9,92E-02	FALSE	-0,46	4,07E-13	1,86E-11	FALSE	1;0
PSMC1	proteasome (prosome, macropain) 26S subunit, ATPase, 1	Hs.356654	-0,51	4,32E-03	1,01E-01	FALSE	-0,16	1,49E-02	5,73E-02	FALSE	1;2
EPB41L3	erythrocyte membrane protein band 4.1-like 3		-0,51	4,40E-03	1,02E-01	FALSE	-0,44	3,47E-12	1,37E-10	FALSE	0;3
COL6A2	collagen, type VI, alpha 2	Hs.420269	-0,51	4,40E-03	1,02E-01	FALSE	-0,22	7,50E-04	5,25E-03	FALSE	1;5
SLC25A21	solute carrier family 25 (mitochondrial oxoadipate carrier), member 21	Hs.730857	-0,51	4,40E-03	1,02E-01	FALSE	-0,30	5,49E-06	7,58E-05	FALSE	1;5
TTLL11	tubulin tyrosine ligase-like family member 11	Hs.438937	-0,50	4,53E-03	1,03E-01	FALSE	-0,21	2,09E-03	1,22E-02	FALSE	0;2
CFTR	cystic fibrosis transmembrane conductance regulator (ATP-binding cassette sub-family C, member 7)	Hs.489786	-0,50	4,53E-03	1,03E-01	FALSE	-0,21	1,44E-03	8,97E-03	FALSE	1;1
GPR160	G protein-coupled receptor 160	Hs.231320	-0,50	4,57E-03	1,03E-01	FALSE	-0,32	1,16E-06	1,85E-05	FALSE	1;2
IL20RA	interleukin 20 receptor, alpha	Hs.445868	-0,50	4,61E-03	1,04E-01	FALSE	-0,24	2,29E-04	1,96E-03	FALSE	0;1
ATG9A	autophagy related 9A	Hs.323363	-0,50	4,78E-03	1,05E-01	FALSE	-0,15	2,10E-02	7,46E-02	FALSE	1;2
HMGN3	high mobility group nucleosomal binding domain 3	Hs.77558	-0,50	4,78E-03	1,05E-01	FALSE	-0,15	2,13E-02	7,54E-02	FALSE	1;2
STAT5B	signal transducer and activator of transcription 5B	Hs.595276	-0,50	4,91E-03	1,08E-01	FALSE	-0,18	5,84E-03	2,73E-02	FALSE	2;1
TRIM31	tripartite motif containing 31	Hs.493275	-0,50	4,96E-03	1,08E-01	FALSE	-0,27	3,27E-05	3,69E-04	FALSE	0;2
SLC6A20	solute carrier family 6 (proline IMINO transporter), member 20	Hs.413095	-0,50	4,96E-03	1,08E-01	FALSE	-0,22	7,94E-04	5,51E-03	FALSE	1;1
ELOVL5	ELOVL fatty acid elongase 5	Hs.520189	-0,50	5,00E-03	1,09E-01	FALSE	-0,52	0,00E+00	0,00E+00	FALSE	1;2
GALNT4	polypeptide N-acetylgalactosaminyltransferase 4	Hs.25130	-0,50	5,14E-03	1,10E-01	FALSE	-0,21	1,39E-03	8,68E-03	FALSE	1;0
EVI2B	ecotropic viral integration site 2B	Hs.5509	-0,50	5,28E-03	1,12E-01	FALSE	-0,27	3,40E-05	3,83E-04	FALSE	0;0
ZNF264	zinc finger protein 264	Hs.515634	-0,50	5,32E-03	1,13E-01	FALSE	-0,61	0,00E+00	0,00E+00	FALSE	1;2
HUNK	hormonally up-regulated Neu-associated kinase	Hs.109437	-0,50	5,37E-03	1,14E-01	FALSE	-0,48	1,64E-14	8,80E-13	FALSE	1,2
GSTM4	glutathione S-transferase mu 4	Hs.348387	-0,49	5,42E-03	1,14E-01	FALSE	-0,22	1,22E-03	7,80E-03	FALSE	1;1
BCAM	basal cell adhesion molecule (Lutheran blood group)	Hs.625725	-0,49	5,47E-03	1,15E-01	FALSE	-0,19	3,92E-03	2,01E-02	FALSE	1;3

Gene ID	Name	UGCluster	Spearman r (cell lines)	Spearman p (cell lines)	FDR adjusted (cell lines)	L-shape (cell lines)	Spearman r (primay tumors)	Spearman p (primay tumors)	FDR adjusted (primay tumors)	L-shape (primay tumors)	CpG islands (promoter;intragenic)[1]
ENTPD2	ectonucleoside triphosphate diphosphohydrolase 2	Hs.123036	-0,49	5,52E-03	1,16E-01	FALSE	-0,25	1,46E-04	1,35E-03	FALSE	1;2
HR	hairless homolog (mouse)		-0,49	5,57E-03	1,16E-01	FALSE	-0,29	1,03E-05	1,34E-04	FALSE	1;1
ZNF615	zinc finger protein 615	Hs.368355	-0,49	5,62E-03	1,17E-01	FALSE	-0,12	7,58E-02	1,92E-01	FALSE	1;1
ITGB7	integrin, beta 7	Hs.654470	-0,49	5,67E-03	1,17E-01	FALSE	-0,23	4,53E-04	3,46E-03	FALSE	0;1
ARHGEF10	Rho guanine nucleotide exchange factor (GEF) 10	Hs.98594	-0,49	5,67E-03	1,17E-01	FALSE	-0,50	1,78E-15	1,08E-13	FALSE	1;8
TFF1	trefoil factor 1	Hs.162807	-0,49	5,92E-03	1,21E-01	FALSE	-0,52	0,00E+00	0,00E+00	FALSE	0;0
IMPDH1	IMP (inosine 5'-monophosphate) dehydrogenase 1	Hs.654401	-0,49	5,92E-03	1,21E-01	FALSE	-0,18	8,55E-03	3,70E-02	FALSE	2;2
NAP1L5	nucleosome assembly protein 1-like 5	Hs.12554	-0,49	5,97E-03	1,21E-01	FALSE	-0,30	6,74E-06	9,14E-05	FALSE	0;1
TM4SF5	transmembrane 4 L six family member 5	Hs.184194	-0,49	5,97E-03	1,21E-01	FALSE	-0,20	2,97E-03	1,61E-02	FALSE	0;1
PRDM5	PR domain containing 5	Hs.666782	-0,49	5,97E-03	1,21E-01	FALSE	-0,23	4,05E-04	3,14E-03	FALSE	1;2
MAPRE3	microtubule-associated protein, RP/EB family, member 3	Hs.515860	-0,49	6,08E-03	1,22E-01	FALSE	-0,31	1,60E-06	2,47E-05	FALSE	0;2
OXCT1	3-oxoacid CoA transferase 1	Hs.278277	-0,49	6,08E-03	1,22E-01	FALSE	-0,42	6,20E-11	2,06E-09	FALSE	1;3
MEP1A	meprin A, alpha (PABA peptide hydrolase)	Hs.179704	-0,49	6,13E-03	1,23E-01	FALSE	-0,29	7,77E-06	1,04E-04	FALSE	0;0
TXNL4B	thioredoxin-like 4B	Hs.134406	-0,49	6,24E-03	1,25E-01	FALSE	-0,13	4,43E-02	1,31E-01	FALSE	0;1
PKP1	plakophilin 1	Hs.497350	-0,49	6,29E-03	1,25E-01	FALSE	-0,30	3,63E-06	5,26E-05	FALSE	1;1
BATF	basic leucine zipper transcription factor, ATF-like	Hs.509964	-0,49	6,35E-03	1,26E-01	FALSE	-0,74	0,00E+00	0,00E+00	FALSE	0;1
CD5	CD5 molecule	Hs.58685	-0,49	6,52E-03	1,28E-01	FALSE	-0,25	2,03E-04	1,78E-03	FALSE	0;0
IFIT3	interferon-induced protein with tetratricopeptide repeats 3	Hs.47338	-0,49	6,52E-03	1,28E-01	FALSE	-0,12	7,42E-02	1,90E-01	FALSE	0;0
FAM111B	family with sequence similarity 111, member B	Hs.186579	-0,49	6,57E-03	1,29E-01	FALSE	-0,21	1,49E-03	9,21E-03	FALSE	2;0
HAAO	3-hydroxyanthranilate 3,4-dioxygenase	Hs.368805	-0,48	6,75E-03	1,30E-01	FALSE	-0,22	9,76E-04	6,53E-03	FALSE	1;1
RUNX1	runt-related transcription factor 1	Hs.149261	-0,48	6,75E-03	1,30E-01	FALSE	-0,28	2,17E-05	2,56E-04	FALSE	1;3
U2AF2	U2 small nuclear RNA auxiliary factor 2	Hs.528007	-0,48	6,80E-03	1,31E-01	FALSE	-0,21	1,53E-03	9,34E-03	FALSE	1;4
PRSS8	protease, serine, 8	Hs.75799	-0,48	6,92E-03	1,32E-01	FALSE	-0,27	5,54E-05	5,88E-04	FALSE	0;0
AADAT	aminoadipate aminotransferase	Hs.529735	-0,48	7,04E-03	1,32E-01	FALSE	-0,12	7,02E-02	1,83E-01	FALSE	1;1
CHAC1	ChaC glutathione-specific gamma-glutamylcyclotransferase 1	Hs.155569	-0,48	7,04E-03	1,32E-01	FALSE	-0,21	1,78E-03	1,06E-02	FALSE	1;1
PNMA2	paraneoplastic Ma antigen 2	Hs.591838	-0,48	7,04E-03	1,32E-01	FALSE	-0,28	3,04E-05	3,47E-04	FALSE	1;1
CDC42EP5	CDC42 effector protein (Rho GTPase binding) 5	Hs.415791	-0,48	7,04E-03	1,32E-01	FALSE	-0,18	8,26E-03	3,61E-02	FALSE	1;2
TMEM106A	transmembrane protein 106A	Hs.536474	-0,48	7,10E-03	1,33E-01	FALSE	-0,58	0,00E+00	0,00E+00	FALSE	1;1
IHH	indian hedgehog	Hs.654504	-0,48	7,16E-03	1,34E-01	FALSE	-0,27	3,81E-05	4,22E-04	FALSE	1;2
ZNF134	zinc finger protein 134	Hs.469694	-0,48	7,22E-03	1,34E-01	FALSE	-0,73	0,00E+00	0,00E+00	FALSE	1;1
TCF7	transcription factor 7 (T-cell specific, HMG-box)	Hs.573153	-0,48	7,35E-03	1,35E-01	FALSE	-0,13	5,72E-02	1,57E-01	FALSE	1;1
CARD11	caspase recruitment domain family, member 11	Hs.648101	-0,48	7,41E-03	1,36E-01	FALSE	-0,62	0,00E+00	0,00E+00	FALSE	0;0
SH3BP1	SH3-domain binding protein 1	Hs.601143	-0,48	7,41E-03	1,36E-01	FALSE	-0,22	8,07E-04	5,60E-03	FALSE	1;1
PLEKHA4	pleckstrin homology domain containing, family A (phosphoinositide binding specific) member 4	Hs.9469	-0,48	7,41E-03	1,36E-01	FALSE	-0,38	6,32E-09	1,54E-07	FALSE	1;2
TFF3	trefoil factor 3 (intestinal)	Hs.82961	-0,48	7,47E-03	1,36E-01	FALSE	-0,17	1,23E-02	4,94E-02	FALSE	0;0
LIMD1	LIM domains containing 1	Hs.193370	-0,48	7,73E-03	1,39E-01	FALSE	-0,31	2,48E-06	3,71E-05	FALSE	1;0
OVOL1	ovo-like zinc finger 1	Hs.134434	-0,48	7,86E-03	1,40E-01	FALSE	-0,14	3,06E-02	9,97E-02	FALSE	1;1
GNA15	guanine nucleotide binding protein (G protein), alpha 15 (Gq class)	Hs.73797	-0,48	7,86E-03	1,40E-01	FALSE	-0,37	1,34E-08	3,07E-07	FALSE	1;2
SLCO3A1	solute carrier organic anion transporter family, member 3A1	Hs.311187	-0,48	7,86E-03	1,40E-01	FALSE	-0,25	1,70E-04	1,54E-03	FALSE	1;2
CAPG	capping protein (actin filament), gelsolin-like	Hs.516155	-0,48	7,86E-03	1,40E-01	FALSE	-0,31	1,98E-06	3,00E-05	FALSE	2;1
RGS10	regulator of G-protein signaling 10	Hs.501200	-0,48	7,93E-03	1,41E-01	FALSE	-0,45	1,26E-12	5,35E-11	FALSE	0;0
NETO2	neuropilin (NRP) and tolloid (TLL)-like 2	Hs.645802	-0,48	7,93E-03	1,41E-01	FALSE	-0,29	1,47E-05	1,81E-04	FALSE	1;1
ADCY3	adenylate cyclase 3	Hs.467898	-0,48	7,93E-03	1,41E-01	FALSE	-0,21	1,28E-03	8,06E-03	FALSE	1;2
ACOT4	acyl-CoA thioesterase 4	Hs.49433	-0,47	7,99E-03	1,41E-01	FALSE	-0,25	1,96E-04	1,73E-03	FALSE	1;1
DDAH2	dimethylarginine dimethylaminohydrolase 2	Hs.247362	-0,47	7,99E-03	1,41E-01	FALSE	-0,44	4,43E-12	1,72E-10	FALSE	1;1
ZNF681	zinc finger protein 681	Hs.399952	-0,47	8,13E-03	1,42E-01	FALSE	-0,46	3,64E-13	1,68E-11	FALSE	1;1
MBD1	methyl-CpG binding domain protein 1	Hs.405610	-0,47	8,13E-03	1,42E-01	FALSE	-0,13	5,78E-02	1,58E-01	FALSE	1;2
LIPH	lipase, member H	Hs.68864	-0,47	8,13E-03	1,42E-01	FALSE	-0,12	7,21E-02	1,86E-01	FALSE	2;1
RPP25	ribonuclease P/MRP 25kDa subunit	Hs.8562	-0,47	8,27E-03	1,43E-01	FALSE	-0,21	1,84E-03	1,09E-02	FALSE	2;1
SLC30A2	solute carrier family 30 (zinc transporter), member 2	Hs.143545	-0,47	8,62E-03	1,47E-01	FALSE	-0,37	1,09E-08	2,53E-07	TRUE	1;1
KLHL21	kelch-like family member 21	Hs.7764	-0,47	8,69E-03	1,48E-01	FALSE	-0,32	1,43E-06	2,23E-05	FALSE	0;0
PSD3	pleckstrin and Sec7 domain containing 3	Hs.434255	-0,47	8,76E-03	1,49E-01	FALSE	-0,16	1,60E-02	6,06E-02	FALSE	0;3
RBP2	retinol binding protein 2, cellular	Hs.655516	-0,47	8,83E-03	1,50E-01	FALSE	-0,52	0,00E+00	0,00E+00	FALSE	0;0
DISP2	dispatched homolog 2 (Drosophila)	Hs.355645	-0,47	8,83E-03	1,50E-01	FALSE	-0,15	2,15E-02	7,59E-02	FALSE	1;2
ID4	inhibitor of DNA binding 4, dominant negative helix-loop-helix protein	Hs.519601	-0,47	8,91E-03	1,50E-01	FALSE	-0,37	1,08E-08	2,52E-07	FALSE	1;1
CYP4F11	cytochrome P450, family 4, subfamily F, polypeptide 11	Hs.187393	-0,47	9,06E-03	1,51E-01	FALSE	-0,49	7,55E-15	4,26E-13	FALSE	0;0
KCNV1	potassium channel, voltage gated modifier subfamily V, member 1	Hs.13285	-0,47	9,13E-03	1,52E-01	FALSE	-0,30	7,06E-06	9,49E-05	TRUE	2;1
KCNH8	potassium channel, voltage gated eag related subfamily H, member 8	Hs.475656	-0,47	9,21E-03	1,52E-01	TRUE	-0,27	4,88E-05	5,25E-04	FALSE	2;6
PRDM16	PR domain containing 16	Hs.99500	-0,47	9,51E-03	1,55E-01	TRUE	-0,29	1,30E-05	1,64E-04	FALSE	2;38
ZNF614	zinc finger protein 614	Hs.292336	-0,46	9,67E-03	1,56E-01	FALSE	-0,38	5,93E-09	1,46E-07	FALSE	1;1
ZNF512	zinc finger protein 512	Hs.529178	-0,46	9,67E-03	1,56E-01	FALSE	-0,51	2,22E-16	1,48E-14	FALSE	1;2
KRT17	keratin 17, type I	Hs.2785	-0,46	9,75E-03	1,56E-01	FALSE	-0,25	1,50E-04	1,38E-03	FALSE	0;0
LRRC34	leucine rich repeat containing 34	Hs.591289	-0,46	9,75E-03	1,56E-01	FALSE	-0,45	1,90E-12	7,87E-11	FALSE	0;1
TRIM2	tripartite motif containing 2	Hs.435711	-0,46	9,75E-03	1,56E-01	FALSE	-0,33	6,56E-07	1,09E-05	FALSE	0;6
IRX5	iroquois homeobox 5	Hs.435730	-0,46	9,75E-03	1,56E-01	FALSE	-0,22	7,16E-04	5,06E-03	FALSE	1;1
LCP1	lymphocyte cytosolic protein 1 (L-plastin)	Hs.381099	-0,46	9,83E-03	1,57E-01	FALSE	-0,38	5,88E-09	1,45E-07	FALSE	0;3
MAP4K2	mitogen-activated protein kinase kinase kinase kinase 2	Hs.534341	-0,46	9,83E-03	1,57E-01	FALSE	-0,45	9,59E-13	4,14E-11	FALSE	1;1
XRCC6	X-ray repair complementing defective repair in Chinese hamster cells 6	Hs.292493	-0,46	9,83E-03	1,57E-01	FALSE	-0,24	2,48E-04	2,08E-03	FALSE	1;2
GSTP1	glutathione S-transferase pi 1	Hs.523836	-0,46	1,01E-02	1,58E-01	FALSE	-0,43	1,26E-11	4,61E-10	FALSE	1;1
IRF8	interferon regulatory factor 8	Hs.137427	-0,46	1,02E-02	1,59E-01	FALSE	-0,18	7,40E-03	3,31E-02	FALSE	1;1
ANXA6	annexin A6	Hs.412117	-0,46	1,02E-02	1,59E-01	FALSE	-0,26	7,41E-05	7,48E-04	FALSE	0;0
SPINT1	serine peptidase inhibitor, Kunitz type 1	Hs.233950	-0,46	1,02E-02	1,59E-01	FALSE	-0,13	5,27E-02	1,48E-01	FALSE	1;1
BCL11A	B-cell CLL/lymphoma 11A (zinc finger protein)	Hs.370549	-0,46	1,03E-02	1,60E-01	FALSE	-0,12	6,30E-02	1,69E-01	FALSE	1;2
HIST1H2BH	histone cluster 1, H2bh	Hs.247815	-0,46	1,12E-02	1,67E-01	FALSE	-0,23	6,89E-04	4,93E-03	FALSE	2;1
MSLN	mesothelin	Hs.408488	-0,46	1,14E-02	1,70E-01	FALSE	-0,22	7,46E-04	5,23E-03	FALSE	0;1
DEF6	differentially expressed in FDCP 6 homolog (mouse)	Hs.15476	-0,46	1,14E-02	1,71E-01	FALSE	-0,16	1,38E-02	5,38E-02	FALSE	0;1
CAB39L	calcium binding protein 39-like	Hs.87159	-0,45	1,15E-02	1,72E-01	FALSE	-0,63	0,00E+00	0,00E+00	FALSE	0;0
CDH1	cadherin 1, type 1, E-cadherin (epithelial)	Hs.461086	-0,45	1,15E-02	1,72E-01	FALSE	-0,18	6,39E-03	2,94E-02	FALSE	1;2
MANSC1	MANSC domain containing 1	Hs.591145	-0,45	1,16E-02	1,72E-01	FALSE	-0,33	3,99E-07	6,92E-06	FALSE	1;1
TPM1	tropomyosin 1 (alpha)	Hs.133892	-0,45	1,16E-02	1,72E-01	FALSE	-0,45	1,43E-12	6,07E-11	FALSE	1;2
SNX16	sorting nexin 16	Hs.492121	-0,45	1,19E-02	1,74E-01	FALSE	-0,12	7,15E-02	1,85E-01	FALSE	0;1
FADS2	fatty acid desaturase 2		-0,45	1,19E-02	1,74E-01	FALSE	-0,47	2,16E-13	1,02E-11	FALSE	2;2
MGST1	microsomal glutathione S-transferase 1	Hs.389700	-0,45	1,21E-02	1,76E-01	FALSE	-0,43	1,57E-11	5,64E-10	FALSE	0;0
MIPOL1	mirror-image polydactyly 1	Hs.660396	-0,45	1,22E-02	1,77E-01	FALSE	-0,46	2,70E-13	1,27E-11	FALSE	1;2
TIMP4	TIMP metallopeptidase inhibitor 4	Hs.591665	-0,45	1,23E-02	1,78E-01	FALSE	-0,24	2,31E-04	1,98E-03	FALSE	0;1
SMPD3	sphingomyelin phosphodiesterase 3, neutral membrane (neutral sphingomyelinase II)	Hs.368421	-0,45	1,24E-02	1,79E-01	FALSE	-0,23	6,57E-04	4,73E-03	FALSE	1;4
GPA33	glycoprotein A33 (transmembrane)	Hs.651244	-0,45	1,25E-02	1,79E-01	FALSE	-0,25	1,56E-04	1,43E-03	FALSE	0;0
PPP1R2	protein phosphatase 1, regulatory (inhibitor) subunit 2	Hs.535731	-0,45	1,25E-02	1,79E-01	FALSE	-0,12	6,53E-02	1,74E-01	FALSE	1;1
NEK7	NIMA-related kinase 7	Hs.24119	-0,45	1,27E-02	1,81E-01	FALSE	-0,13	4,73E-02	1,37E-01	FALSE	1;5
ACSS1	acyl-CoA synthetase short-chain family member 1	Hs.529353	-0,45	1,28E-02	1,82E-01	FALSE	-0,26	8,34E-05	8,31E-04	FALSE	0;0
ECE1	endothelin converting enzyme 1	Hs.195080	-0,45	1,28E-02	1,82E-01	FALSE	-0,19	3,74E-03	1,93E-02	FALSE	0;3
MRPL19	mitochondrial ribosomal protein L19	Hs.44024	-0,45	1,31E-02	1,84E-01	FALSE	-0,13	5,00E-02	1,42E-01	FALSE	0;1

Gene ID	Name	UGCluster	Spearman r (cell lines)	Spearman p (cell lines)	FDR adjusted (cell lines)	L-shape (cell lines)	Spearman r (primay tumors)	Spearman p (primay tumors)	FDR adjusted (primay tumors)	L-shape (primay tumors)	CpG islands (promoter;intragenic)[1]
TMEM125	transmembrane protein 125	Hs.104476	-0,45	1,31E-02	1,84E-01	FALSE	-0,12	7,66E-02	1,94E-01	FALSE	0;1
MLF2	myeloid leukemia factor 2	Hs.524214	-0,45	1,31E-02	1,84E-01	FALSE	-0,20	2,90E-03	1,58E-02	FALSE	2;2
PLEK2	pleckstrin 2	Hs.170473	-0,45	1,33E-02	1,86E-01	FALSE	-0,13	4,95E-02	1,42E-01	FALSE	1;1
FZD10	frizzled class receptor 10	Hs.31664	-0,45	1,34E-02	1,87E-01	FALSE	-0,51	2,22E-16	1,48E-14	FALSE	1;1
ZC3HAV1	zinc finger CCCH-type, antiviral 1	Hs.133512	-0,45	1,36E-02	1,89E-01	FALSE	-0,15	3,00E-02	9,82E-02	FALSE	1;5
ICA1	islet cell autoantigen 1, 69kDa	Hs.487561	-0,45	1,37E-02	1,90E-01	FALSE	-0,21	1,31E-03	8,22E-03	FALSE	1;1
NMNAT2	nicotinamide nucleotide adenylyltransferase 2	Hs.497123	-0,45	1,37E-02	1,90E-01	FALSE	-0,41	2,11E-10	6,55E-09	FALSE	1;2
COL27A1	collagen, type XXVII, alpha 1	Hs.494892	-0,44	1,38E-02	1,91E-01	FALSE	-0,30	4,10E-06	5,80E-05	TRUE	0;0
RHOH	ras homolog family member H	Hs.654594	-0,44	1,40E-02	1,92E-01	FALSE	-0,49	7,33E-15	4,15E-13	FALSE	0;1
LAD1	ladinin 1	Hs.519035	-0,44	1,40E-02	1,92E-01	FALSE	-0,15	2,27E-02	7,94E-02	FALSE	1;1
PROM2	prominin 2	Hs.437376	-0,44	1,41E-02	1,93E-01	FALSE	-0,77	0,00E+00	0,00E+00	FALSE	0;0
RAD51C	RAD51 paralog C	Hs.412587	-0,44	1,41E-02	1,93E-01	FALSE	-0,19	4,41E-03	2,20E-02	FALSE	1;1
ELOVL3	ELOVL fatty acid elongase 3	Hs.302130	-0,44	1,42E-02	1,94E-01	FALSE	-0,22	9,26E-04	6,26E-03	FALSE	1;1
APOL6	apolipoprotein L, 6	Hs.257352	-0,44	1,43E-02	1,94E-01	FALSE	-0,24	2,63E-04	2,18E-03	FALSE	0;0
EFNA5	ephrin-A5	Hs.288741	-0,44	1,45E-02	1,95E-01	FALSE	-0,17	1,03E-02	4,29E-02	FALSE	1;2
CACNB2	calcium channel, voltage-dependent, beta 2 subunit	Hs.59093	-0,44	1,46E-02	1,96E-01	FALSE	-0,17	1,17E-02	4,72E-02	FALSE	1;3
GNPTG	N-acetylglucosamine-1-phosphate transferase, gamma subunit	Hs.241575	-0,44	1,46E-02	1,96E-01	FALSE	-0,13	4,66E-02	1,36E-01	FALSE	2;4
FANCF	Fanconi anemia, complementation group F	Hs.632151	-0,44	1,48E-02	1,97E-01	FALSE	-0,20	2,72E-03	1,51E-02	FALSE	1;1
RHOD	ras homolog family member D	Hs.15114	-0,44	1,50E-02	1,99E-01	FALSE	-0,52	0,00E+00	0,00E+00	FALSE	1;2
CPNE7	copine VII	Hs.461775	-0,44	1,50E-02	1,99E-01	FALSE	-0,29	9,79E-06	1,28E-04	FALSE	2;7
THRB	thyroid hormone receptor, beta	Hs.187861	-0,42	2,05E-02	2,35E-01	TRUE	-0,34	2,88E-07	5,13E-06	FALSE	1;2
MCC	mutated in colorectal cancers	Hs.593171	-0,37	4,56E-02	3,41E-01	TRUE	-0,16	1,87E-02	6,84E-02	FALSE	1;4
ST6GALNAC3	ST6 (alpha-N-acetyl-neuraminyl-2,3-beta-galactosyl-1,3)-N-acetylgalactosaminide alpha-2,6-sialyltransferase 3	Hs.337040	-0,35	5,63E-02	3,78E-01	TRUE	-0,12	6,40E-02	1,71E-01	FALSE	1;3
MYRIP	myosin VIIA and Rab interacting protein	Hs.594535	-0,35	5,90E-02	3,87E-01	TRUE	-0,34	1,87E-07	3,47E-06	FALSE	0;4
MYT1	myelin transcription factor 1	Hs.279562	-0,29	1,17E-01	5,17E-01	TRUE	-0,24	2,37E-04	2,02E-03	FALSE	0;3
CD200	CD200 molecule	Hs.79015	-0,29	1,17E-01	5,19E-01	TRUE	-0,24	2,52E-04	2,11E-03	FALSE	2;2

[1] CpG islands as defined by Gardiner-Garden and Frommer (DOI: 10.1016/0022-2836(87)90689-9). Promoter;Intragenic CpG island

Supplementary Table S3: Functional group enrichement analysis carried out with DAVID (https://doi.org/10.1186/gb-2003-4-9-r60).

Category	Term	Count	%	Entrez Gene ID of genes in category	List Total	Pop Hits	Pop Total	Fold Enrichment	PValue	FDR
UP_SEQ_FEATURE	zinc finger region:C2H2-type 4	33	8,64	126231, 7700, 130557, 84450, 11107, 10172, 53335, 59348, 146434, 90233, 147923, 5017, 79088, 7766, 79027, 348327, 5325, 162972, 84924, 55205, 9422, 63976, 7762, 91392, 199704, 27300, 79898, 25850, 10472, 284370, 80110, 7693, 58491	381	261	9840	3,3	5,78E-09	9,25E-06
UP_SEQ_FEATURE	zinc finger region:C2H2-type 8	27	7,07	126231, 7700, 130557, 11107, 10172, 59348, 90233, 147923, 79088, 7766, 348327, 162972, 148213, 84924, 55205, 7561, 63976, 9422, 91392, 199704, 27300, 79898, 25850, 284370, 80110, 7693, 58491	381	187	9840	3,7	1,21E-08	1,93E-05
UP_SEQ_FEATURE	zinc finger region:C2H2-type 6	29	7,59	126231, 7700, 130557, 11107, 10172, 53335, 59348, 146434, 90233, 147923, 79088, 79027, 348327, 5325, 162972, 84924, 55205, 7561, 63976, 9422, 91392, 199704, 27300, 79898, 25850, 284370, 80110, 7693, 58491	381	216	9840	3,5	1,58E-08	2,53E-05
UP_SEQ_FEATURE	zinc finger region:C2H2-type 3	34	8,90	126231, 7700, 130557, 10172, 53335, 59348, 55809, 146434, 90233, 147923, 5017, 79088, 7766, 79027, 348327, 5325, 162972, 84924, 55205, 7561, 9422, 63976, 8609, 7762, 91392, 199704, 27300, 79898, 25850, 10472, 284370, 80110, 7693, 58491	381	294	9840	3,0	3,00E-08	4,81E-05
UP_SEQ_FEATURE	zinc finger region:C2H2-type 5	30	7,85	126231, 7700, 130557, 11107, 10172, 53335, 59348, 146434, 90233, 147923, 79088, 7766, 79027, 348327, 5325, 162972, 84924, 55205, 7561, 63976, 9422, 91392, 199704, 27300, 79898, 25850, 284370, 80110, 7693, 58491	381	237	9840	3,3	3,22E-08	5,16E-05
UP_SEQ_FEATURE	zinc finger region:C2H2-type 11	21	5,50	126231, 7700, 55205, 7561, 11107, 9422, 10172, 91392, 199704, 27300, 90233, 79898, 25850, 147923, 284370, 79088, 80110, 7693, 348327, 58491, 148213	381	123	9840	4,4	4,32E-08	6,92E-05
UP_SEQ_FEATURE	zinc finger region:C2H2-type 7	27	7,07	126231, 7700, 130557, 11107, 10172, 59348, 146434, 90233, 147923, 79088, 7766, 348327, 5325, 162972, 148213, 84924, 55205, 7561, 9422, 91392, 27300, 79898, 25850, 284370, 80110, 7693, 58491	381	202	9840	3,5	6,06E-08	9,70E-05
UP_SEQ_FEATURE	zinc finger region:C2H2-type 12	19	4,97	126231, 55205, 7561, 11107, 9422, 10172, 91392, 199704, 27300, 90233, 79898, 25850, 147923, 79088, 284370, 80110, 348327, 58491, 148213	381	110	9840	4,5	1,87E-07	3,00E-04
UP_SEQ_FEATURE	zinc finger region:C2H2-type 2	32	8,38	126231, 7700, 130557, 84450, 10172, 53335, 59348, 55809, 146434, 90233, 5017, 147923, 79088, 7766, 79027, 348327, 5325, 162972, 148213, 84924, 63976, 9422, 8609, 7762, 91392, 199704, 27300, 79898, 25850, 10472, 284370, 58491	381	287	9840	2,9	1,95E-07	3,12E-04
UP_SEQ_FEATURE	zinc finger region:C2H2-type 9	23	6,02	126231, 7700, 55205, 7561, 11107, 9422, 63976, 10172, 91392, 199704, 27300, 90233, 79898, 25850, 147923, 284370, 79088, 80110, 7766, 7693, 348327, 58491, 148213	381	162	9840	3,7	2,51E-07	4,01E-04
UP_SEQ_FEATURE	zinc finger region:C2H2-type 10	21	5,50	126231, 7700, 7561, 11107, 9422, 63976, 10172, 91392, 199704, 27300, 90233, 79898, 25850, 147923, 284370, 79088, 80110, 7693, 348327, 58491, 148213	381	140	9840	3,9	3,88E-07	6,20E-04
UP_SEQ_FEATURE	domain:KRAB	20	5,24	126231, 84924, 7700, 7561, 9422, 10172, 7762, 59348, 199704, 27300, 146434, 90233, 79898, 147923, 284370, 79088, 80110, 7766, 348327, 162972	381	132	9840	3,9	6,75E-07	0,00107986
UP_SEQ_FEATURE	zinc finger region:C2H2-type 1	29	7,59	126231, 130557, 84450, 11107, 10172, 53335, 59348, 55809, 146434, 90233, 5017, 147923, 7766, 79027, 348327, 5325, 162972, 7561, 9422, 8609, 7762, 91392, 199704, 79898, 25850, 10472, 284370, 7693, 58491	381	266	9840	2,8	1,30E-06	0,00207941
UP_SEQ_FEATURE	zinc finger region:C2H2-type 13	15	3,93	126231, 7561, 11107, 9422, 10172, 91392, 199704, 27300, 90233, 25850, 147923, 284370, 348327, 58491, 148213	381	83	9840	4,7	3,01E-06	0,00482106
UP_SEQ_FEATURE	zinc finger region:C2H2-type 14	11	2,88	126231, 25850, 147923, 7561, 284370, 11107, 10172, 91392, 148213, 199704, 90233	381	62	9840	4,6	1,17E-04	0,18696963

Supplementary Table S4: Clinicopathological features of the 132 Dukes ' C colorectal cancer patients in this study as a funtion of ZBTB 18 protein expression as determined by immunohistochemistry.

	Total	Low ZBTB18	High ZBTB18	p value
**Sex, n (%)**				
Female	65 (49.3)	53 (47.4)	12 (60)	0.33[1]
Male	67 (50.7)	59 (52.6)	8 (40)	
**Age (years), mean±SD**	66,7	66.6+/-12.5	66.9+/-11.7	0.91[2]
**Site, n (%)**				
Colon	78 (59.6)	64 (57.7)	14 (70)	0.33[1]
Rectum	53 (40.4)	47 (42.3)	6 (30)	
**Degree of diffirentiation, n (%)**				
Good	13 (10)	11 (10)	2 (10)	0.90[1]
Moderate	100 (76.9)	84 (76.3)	16 (80)	
Poor	17 (13)	15 (13.6)	2 (10)	
**5-year overall survival, n (%)**				
Alive	56 (42.5)	42 (37.5)	14 (70)	0.01[1]
Dead	76 (57.5)	70 (62.5)	6 (30)	
**5-year disease-free survival, n (%)**				
Alive	49 (40.2)	37 (36.3)	12 (60)	0.07[1]
Dead	73 (59.8)	65 (63.7)	8 (40)	
**Adjuvant treatment, n (%)**				
Yes	54 (44)	48 (45.8)	6 (33.4)	0.44[1]
No	69 (56)	57 (54.2)	12 (66.6)	
**Microsatellite instability, n (%)**				
MSI	16 (12.3)	12 (10.9)	4 (20)	0.26[1]
MSS	115 (87.7)	99 (89.1)	16 (80)	
**TP53 status, n (%)**				
Wild type	18 (46.2)	13 (42)	5 (62.5)	0.43[1]
Mutant	21 (53.8)	18 (58)	3 (37.5)	
**KRAS starus**				
Wild type	32 (65.4)	26 (66.7)	6 (60)	0.72[1]
Mutant	17 (34.6)	13 (33.3)	4 (40)	
**Allelic loss of chromosome 18q, n (%)**				
LOH	39 (43.9)	35 (44.4)	4 (40)	1[1]
No LOH	50 (56.1)	44 (55.6)	6 (60)	

[1]Fisher test; [2]Mann Whitney test

Supplementary Table S5: Clinicopathological features of the 55 colorectal Stage III cancer patients from the TCGA as a function of ZBTB 18 mRNA expression as determined by microarray analysis (Agilent).

	Total	Low ZNF238	High ZNF238	p value
**Sex, n (%)**				
Female	31 (56.4)	11 (61.2)	20 (54.1)	0.77[1]
Male	24 (43.6)	7 (38.8)	17 (45.9)	
**Age (years), mean±SD**	68,31	70.3+/-10.0	67.3+/-12.8	0.40[2]
**Site, n (%)**				
Colon	39 (71)	14 (77.8)	25 (67.6)	0.54[1]
Rectum	16 (29)	4 (22.2)	12 (32.4)	
**Mean follow up (years), mean±SD**	1,43	0.94+/-1.03	1.62+/-1.06	0.40[1]
**2-year overall survival, n (%)**				
Alive	22 (71)	4 (36.4)	18 (90)	0.003[1]
Dead	9 (29)	7 (63.6)	2 (10)	
**MS status**				
MSS	48 (87.3)	17 (94.5)	31 (83.8)	0.40[1]
MSI	7 (12.7)	1 (5.5)	6 (16.2)	
**TP53 status**				
WT	18 (34.7)	6 (35.3)	12 (34.3)	1[1]
MUT	34 (65.3)	11 (64.7)	23 (65.7)	
**KRAS status**				
WT	33 (63.5)	12 (70.6)	21 (60)	0.55[1]
MUT	19 (36.5)	5 (29.4)	14 (40)	

[1]Fisher test; [2]Mann Whitney test

Identification of ZBTB18 as a novel colorectal tumor suppressor gene through genome-wide promoter hypermethylation analysis

Sarah Bazzocco[1*], Higinio Dopeso[1*], Águeda Martínez-Barriocanal[1,8*], Estefanía Anguita[1], Rocío Nieto[1], Jing Li[1], Elia García-Vidal[1], Valentina Maggio[1], Paulo Rodrigues[1], Priscila Guimarães de Marcondes[1], Simo Schwartz Jr[2,3], Lauri A. Aaltonen[4], Alex Sánchez[5], John M. Mariadason[6,7], Diego Arango[1,8]

SUPPLEMENTARY MATERIALS AND METHODS

Cell lines and primary tumors. A total of 45 colorectal cancer cell lines were used: CACO2, COLO201, COLO205, COLO320, DLD1, HCT116, HCT15, HT29, LOVO, LS1034, LS174T, LS513, RKO, SKCO1, SW1116, SW403, SW48, SW620, SW837, SW948 and T84, were purchased from ATCC (Manassas, VA). HDC108, HDC111, HDC114, HDC15, HDC75, HDC87 and HDC9 were a kind gift from Dr. Johannes Gebert (Institute of Pathology, University Hospital Heidelberg, Heidelberg, Germany). HCC2998, KM12, RW7213, and RW2982, were a kind gift from Dr. L.H. Augenlicht (Albert Einstein Cancer Center, Bronx, NY, USA). LIM1215 and LIM2405 were obtained from the Ludwig Institute for Cancer Research in Melbourne. ALA, CO115, FET, IS1, IS2, IS3, V9P, and TC71 were a kind gift from Dr. Hamelin (Centre De Recherche Scientifique Saint-Antoine, Paris, France). GP5D, HCA7, and VACO5 were from Dr. L.A. Aaltonen (Biomedicum Helsinki, Finland). All lines were maintained in MEM (Life Technologies, Carlsbad, CA) supplemented with 10% fetal bovine serum, 1x antibiotic/antimycotic (100 units/ml streptomycin, 100 units/ml penicillin, and 0.25 µg/ml amphotericin B), 1x MEM Non-Essential Amino Acids Solution, and 10 mM HEPES buffer solution (all from Life Technologies, Carlsbad, CA) at 37ºC and 5% CO_2. All lines were tested to be negative for mycoplasma contamination (PCR Mycoplasma Detection Set, Takara). Cell lines were cultured until they reached 70%-80% confluence and the medium was changed 8h before harvesting for DNA or RNA extraction. Possible cell line cross-

contamination was ruled out by clustering analysis of genome-wide mRNA expression and promoter methylation data at the time of these experiments.

ZBTB18 overexpression in colon cancer cells. The plasmid pENTR233-ZBTB18 (PlasmID Repository, Harvard Medical School; Clone ID HsCD00376023; NM_006352.3) was used to obtain the ZBTB18 coding sequence. ZBTB18 was PCR-amplified with Phusion High-Fidelity DNA Polymerase (Thermo Fisher Scientific, Waltham, MA), using a forward primer containing the sequence of a BamHI restriction site and a Kozak sequence, and a reverse primer containing also a BamHI restriction sequence (**Supplementary Table 1**). The PCR product was cloned into the BamHI site of the pIRES2-EGFP vector (Clontech, Mountain View, CA), and the resulting pZBTB18-IRES-EGFP plasmid was sequenced verified. HCT116 cells were transfected with pIRES2-EGFP or pZBTB18-IRES-EGFP with Lipofectamine 2000 (Thermo Fisher Scientific, Waltham, MA) according to manufacturer's instructions. For tetracycline-inducible overexpression of ZBTB18 into HT29 cells, the ZBTB18-IRES-EGFP cassette and the control IRES-EGFP cassette were PCR amplified from pZBTB18-IRES-EGFP (**Supplementary Table 1**), cloned into pDONR221 (Invitrogen) and then recombined into the lentiviral Gateway vector pINDUCER20 [1]. HT29 cells were transduced with pINDUCER20-ZBTB18-IRES-EGFP or the empty vector pINDUCER20-IRES-EGFP as previously described [2]. After neomycin selection, EGPF-positive cells in HCT116 derivative cell sublines or in HT29 sublines treated with 1 µg/mL doxycycline, were FACS sorted (FACSAria BD, Franklin Lakes, NJ) to obtain cultures with >87% EGFP positive cells.

Determination of cell growth. To assess the role of ZBTB18 on the growth of colon cancer cells, $3x10^4$ HT29, HCT116 or derivative cells were seeded in triplicate wells of seven 24-well plates and allowed to adhere overnight. Cells were trypsinized and stained with trypan blue at the indicated times at times (0, 24, 48, 72, 96, 120 or 144h) to quantify the number of viable cells using a hemocytometer. Growth curves presented were plotted with Prism (GraphPad Software) and are the average of three independent experiments carried out in triplicate.

Migration assays. To investigate changes in the motility of HCT116 and HT29 cells upon ZBTB18 upregulation, wound-healings assays were conducted. Cells were seeded (1×10^6 and 1.5×10^6 cells, respectively) in 12-well plates and allowed to grow for 24h to

reach confluence. The cell monolayer was scratched with a sterile micropipette tip and the wound region was allowed to heal. The percentage of wound closed as a result of cell migration at 0, 12, 24, 36 and 48h was quantified with Image J software.

Invasion assays. Changes in the invasive properties of cells upon overexpression of ZBTB18 were assessed using 24-well Boyden chambers covered with 10% Matrigel. HCT116 and HT29 cancer cells were seeded (5×10^4 and 1×10^5 cells, respectively) in 100µl of cell culture medium containing 1% FBS in the upper compartment of the transwell. The lower compartment was filled with culture medium with 10% FBS, acting as chemoattractant. After incubation for 48h at 37°C in 5% CO2, the cells that were unable to penetrate the filter were wiped out with a cotton swab, whereas the cells that had invaded the lower surface of the filter were fixed and stained with 5% crystal violet. The total number of invading cells was determined under the microscope.

Determination of cell cycle and apoptosis. Cell cycle and hypodiploid (subG1) cells were quantified by flow cytometry as follows. Cells were washed with PBS, fixed in cold 70% ethanol, permeabilized with a buffer containing sodium citrate and Triton X-100 and then stained with propidium iodide while treated with RNase A. Quantitative analysis was carried out in a FACSCalibur cytometer using Cell Quest software (BD Biosciences).

Quantification of mRNA expression. The levels of expression of more than 47,000 transcripts and variants, including more than 38,500 well characterized genes and UniGenes, were investigated using GeneChip Human Genome U133 Plus 2.0 Array (Affymetrix, Santa Clara, CA) in a subset of 30 colorectal cell lines as previously described (CACO2, CO115, COLO201, COLO205, COLO320, DLD1, HCC2998, HCT116, HCT15, HT29, IS1, IS2, KM12, LIM1215, LIM2405, LOVO, LS174T, RKO, RW2982, RW7213, SKCO1, SW1116, SW403, SW48, SW620, SW837, SW948, T84, TC71 and VACO5) [3]. The mRNA expression microarray obtained data was deposited at ArrayExpress (E-MTAB-2971). Gene expression data for a cohort of 223 primary colorectal tumors (Agilent 244K Custom Gene Expression G4502A-07-1/2/3) was available from The Cancer Genome Atlas (TCGA) [4]. For Real-Time RT-PCR quantification of mRNA expression, cell cultures were harvested at 70% confluence

and total RNA was extracted using the TRI Reagent (Molecular Research Center Inc., Cincinnati, OH) according to the manufacturer's instructions. Total RNA was reverse transcribed using the High Capacity cDNA Reverse Transcription kit (Applied Biosystems, Branchburg, NJ), and relative mRNA levels of ZBTB18, ITGA9, STK33, PPP1R14D, BST2, ZNF550, KLHL3, DPYSL3, ATP6V1C2 and CYP4X1 were assessed by Real-Time PCR using SYBR Green Master Mix (Applied Biosystems, Branchburg, NJ). We used 18S rRNA (Taqman Master Mix) as a standardization control for the ΔΔCt method as described before [3]. The sequence of the primers used is available in **Supplementary Table 1**.

Assessment of DNA methylation. We assessed the quantitative levels of methylation at the single nucleotide resolution level for a total of 27,578 highly informative CpG sites using HumanMethylation27 Beadchip (Illumina, San Diego, CA). These chips target CpG sites located within the proximal promoter regions of transcription start sites of 14,475 consensus coding sequencing (CCDS) in the NCBI Database. The levels of DNA methylation were studied in 45 different lines (ALA, CACO2, CO115, COLO201, COLO205, COLO320, DLD1, FET, GP5D, HCA7, HCC2998, HCT116, HCT15, HDC108, HDC111, HDC114, HDC15, HDC75, HDC87, HDC9, HT29, IS1, IS2, IS3, KM12, LIM1215, LIM2405, LOVO, LS1034, LS174T, LS513, RKO, RW2982, RW7213, SKCO1, SW1116, SW403, SW48, SW620, SW837, SW948, T84, TC71, V9P and VACO5). In addition, one of the lines (SW48) was hybridized twice. Also, an *in vitro* methylated control (CpG Methylated Jurkat Genomic DNA, New England BioLabs, Ipswich, MA) and an unmethylated control (Jurkat DNA amplified *in vitro* with illustra GenomiPhi HY DNA Amplification Kit from GE Healthcare, Chicago, IL) were included in the experimental design. The DNA from all samples was extracted using GenElute Mammalian Genomic DNA Miniprep Kit (Sigma-Aldrich, St. Louis, MO) and then bisulfite treated and hybridized following manufacturer's recommendations at the Spanish National genotyping Center (CeGen-CRG Genotyping Unit). The levels of methylation were calculated using GenomeStudio software (Illumina), and expressed as beta-values (the ratio of the methylated probe intensity and the overall intensity - *i.e.*, sum of methylated and unmethylated probe intensities). Methylation data for this panel of 45 colorectal cancer cell lines was deposited at ArrayExpress (MTAB-7867; released upon

publication). Promoter methylation data for a cohort of 223 primary colorectal tumors (Illumina HumanMethylation27 Beadchip) was available from The Cancer Genome Atlas (TCGA) [4]. For bisulfite sequencing analysis, genomic DNA was bisulfite treated with EZ DNA Methylation-Gold Kit (Zymo Research, Irvine, CA) following the manufacturer's instructions and then PCR-amplified with the following conditions: 95°C for 5 min, then 40 cycles of 94°C for 30 seconds, 59.1°C (ITGA9 and PPP1R14D), 60.4°C (ZBTB18) or 64.1°C (KLHL3) for 45 seconds and 72°C for 30 seconds, and then a final elongation step at 72°C for 10 min. Primers for DNA amplification and sequencing were designed using MethPrimer software [5] and are listed in **Supplementary Table 1**. The amplified regions were sequenced using the Macrogen sequencing facility (Macrogen Inc, Korea).

Associations between mRNA expression and promoter methylation levels. Transcriptomic (Affymetrix Human Genome U133 Plus 2.0 Arrays) and methylomic (Illumina HumanMethylation27 Beadchip) data was available for 30 colorectal cancer cell lines (CACO2, CO115, COLO201, COLO205, COLO320, DLD1, HCC2998, HCT116, HCT15, HT29, IS1, IS2, KM12, LIM1215, LIM2405, LOVO, LS174T, RKO, RW2982, RW7213, SKCO1, SW1116, SW403, SW48, SW620, SW837, SW948, T84, TC71 and VACO5). In addition, transcriptomic (Agilent 244K Custom Gene Expression G4502A-07-1/2/3) and methylomic (Illumina HumanMethylation27 Beadchip) for a cohort of 223 primary colorectal tumors was available from The Cancer Genome Atlas (TCGA) [4]. Using the gene symbol as a common identifier, we found that expression data was available for a total of 11,359 (81.92%) of the 14,475 promoters interrogated in the HumanMethylation27 arrays. For genes with more than one probe the average value of expression/methylation was used. Significant correlations between gene expression and promoter methylation were identified using Spearman correlations (False Discovery Rate (FDR) <0.2). Genes showing a non-linear negative association between expression and methylation levels (i.e., 'L-shaped' in the scattered plots; see **Supplementary Figure 5**) were identified using the *ad hoc* generated tool available at http://cinna.upc.edu:3838/alex/Lheuristic/ (more details can be found in [6]). Genes with CpG islands across the human genome were identified using dbCGI [7].

Gene set enrichment analysis. To investigate whether there was an enrichment in the number of genes with significant correlation between expression and methylation levels that belonged to different categories of functionally related genes, we used the Database for Annotation, Visualization and Integrated Discovery (DAVID) v6.7 [8]. This Functional Classification Tool generates a gene-to-gene similarity matrix based on shared functional annotation using over 75,000 terms from 14 functional annotation sources. A Fisher's exact test was used to identify significantly enriched categories in the 382 genes regulated by methylation relative to the 9,876 genes investigated. A false discovery rate (FDR) below 0.2 was used to correct for multiple hypothesis testing.

Subcutaneous xenograft mouse model. Mice were maintained under sterile conditions and all the experiments were carried out under observance of the protocol approved by the Ethical Committee for Animal Experimentation from the Vall d'Hebron University Hospital Institute of Research (Barcelona, Spain). NOD/SCID mice (6-7 weeks-old) were purchased from Charles River (Wilmington, MA). The tumors were established by subcutaneous injection of $2.5x10^6$ HCT116 derivative cells or $1x10^6$ HT29 derivative cells resuspended in 100µl sterile PBS. Empty vector (EV) control cells and ZBTB18-overexpressing cells were injected in the right and left flank, respectively. All animals bearing tumors from HT29 derivative cells received 1 mg/ml doxycycline and 2.5% sucrose (Sigma) in the drinking water. The long (L) and short (S) axis of the tumors were measured with a caliper three times per week. The tumor volume was calculated using the formula: $V=LxS^2x0.52$. After euthanization of the animals the tumors were resected and the tumor weight was determined before being processed for routine histological analysis.

Tissue microarray analysis. A tissue microarray containing 30 colorectal cancer cell lines use in the study was generated. Briefly, cells were grown for 72h, scrapped from plates, fixed with formalin and embedded in low melt agarose and paraffin, sequentially. Three different punches from each cell line paraffin block were arranged using a Minicore semi-automatic tissue arrayer (Alphelys, France). The tissue microarray from colorectal cancer patients was generated and described previously [9]. For immunohistochemical analysis, the commercial NovoLink polymer detection

system (Novocastra Laboratories, Newcastle, UK) was used according to the instructions of the manufacturer. For ZBTB18 detection, a rabbit polyclonal anti-ZBTB18 antibody was used at 1:1000 dilution (Biorbyt, Cambridge, UK; cat# orb357631). ZBTB18 expression was assessed blinded to the sample identity in 30 colorectal cancer cell lines, 75 samples of normal colon tissues, 132 primary tumors and 15 lymph node metastases, all of them in triplicate. All primary tumors samples were enriched in tumor cells (>70%). All patient samples were collected at diagnosis. ZBTB18 staining intensity in the epithelial compartment of primary tumors was scored using a semiquantitative scale ranging from 0 (absence of ZBTB18 immunostaining) to 3 (high immunostaining). Based on the average ZBTB18 staining value of the primary tumor, patients were dichotomized as high or low ZBTB18. The optimal cutoff value was identified using the 'Cutoff finder' R Package [10]. ZBTB18 staining intensity in the colorectal cancer cell line TMA was scored using QuPath software.

REFERENCES

1. Meerbrey KL, Hu G, Kessler JD, Roarty K, Li MZ, Fang JE, Herschkowitz JI, Burrows AE, Ciccia A, Sun T, et al. The pINDUCER lentiviral toolkit for inducible RNA interference in vitro and in vivo. Proc Natl Acad Sci 2011; 108: 3665–3670.
2. Andretta E, Cartón-García F, Martínez-Barriocanal Á, de Marcondes PG, Jimenez-Flores LM, Macaya I, Bazzocco S, Bilic J, Rodrigues P, Nieto R, et al. Investigation of the role of tyrosine kinase receptor EPHA3 in colorectal cancer. Sci Rep 2017; 7: 41576.
3. Bazzocco S, Dopeso H, Carton-Garcia F, Macaya I, Andretta E, Chionh F, Rodrigues P, Garrido M, Alazzouzi H, Nieto R, et al. Highly Expressed Genes in Rapidly Proliferating Tumor Cells as New Targets for Colorectal Cancer Treatment. Clin Cancer Res 2015; 21: 3695–3704.
4. Cancer Genome Atlas Network. Comprehensive molecular characterization of human colon and rectal cancer. Nature 2012; 487: 330–7.
5. Li L-CC, Dahiya R. MethPrimer: designing primers for methylation PCRs. Bioinformatics 2002; 18: 1427–1431.
6. Sánchez A, Miró B, Castellano P, Carmona F, Bazzocco S, Arango D. A heuristic algorithm to select L-shaped genes, potentially regulated by methylation. Submitted 2020.
7. Yalcin D, Otu HH. Comparative analysis of human and mouse CpG islands using dbCGI. In: IEEE International Conference on Electro Information Technology. 2017, pp 211–216.
8. Huang da W, Sherman BT, Lempicki RA. Systematic and integrative analysis of large gene lists using DAVID bioinformatics resources. Nat Protoc 2009; 4: 44–57.
9. Arango D, Laiho P, Kokko A, Alhopuro P, Sammalkorpi H, Salovaara R, Nicorici D, Hautaniemi S, Alazzouzi H, Mecklin J-P, et al. Gene-expression profiling predicts recurrence in Dukes' C colorectal cancer. Gastroenterology 2005; 129: 874–84.

10. Budczies J, Klauschen F, Sinn B V., Gyorffy B, Schmitt WD, Darb-Esfahani S, Denkert C. Cutoff Finder: A Comprehensive and Straightforward Web Application Enabling Rapid Biomarker Cutoff Optimization. PLoS One 2012; 7: e51862.
